# Phase separation of DUX family proteins drives totipotent-like state via 3D genome reorganization and retrotransposon activation

**DOI:** 10.1093/procel/pwag014

**Published:** 2026-03-15

**Authors:** Leilei Gao, Qifeng Gao, Na Hai, Ziqiang Wu, Penghui Li, Han Kang, Xiaohui Song, Jinlian Hua, Shiqiang Zhang, Gang Ren, Jihong Yang, Leqian Yu, Yulei Wei, Junjun Ding, Fan Yang

**Affiliations:** College of Veterinary Medicine, Northwest A&F University, Yangling 712100, China; Shaanxi Centre of Stem Cells Engineering & Technology, Northwest A & F University, Yangling 712100, China; Key Laboratory of Livestock Biology, Northwest A&F University, Yangling 712100, China; College of Veterinary Medicine, Northwest A&F University, Yangling 712100, China; Shaanxi Centre of Stem Cells Engineering & Technology, Northwest A & F University, Yangling 712100, China; Key Laboratory of Livestock Biology, Northwest A&F University, Yangling 712100, China; State Key Laboratory of Animal Biotech Breeding, College of Biological Sciences, China Agricultural University, Beijing 100193, China; RNA Biomedical Institute, Sun Yat-Sen Memorial Hospital, Zhongshan School of Medicine, Sun Yat-Sen University, Guangzhou 510080, China; RNA Biomedical Institute, Sun Yat-Sen Memorial Hospital, Zhongshan School of Medicine, Sun Yat-Sen University, Guangzhou 510080, China; College of Veterinary Medicine, Northwest A&F University, Yangling 712100, China; Shaanxi Centre of Stem Cells Engineering & Technology, Northwest A & F University, Yangling 712100, China; Key Laboratory of Livestock Biology, Northwest A&F University, Yangling 712100, China; College of Veterinary Medicine, Northwest A&F University, Yangling 712100, China; Shaanxi Centre of Stem Cells Engineering & Technology, Northwest A & F University, Yangling 712100, China; Key Laboratory of Livestock Biology, Northwest A&F University, Yangling 712100, China; College of Veterinary Medicine, Northwest A&F University, Yangling 712100, China; Shaanxi Centre of Stem Cells Engineering & Technology, Northwest A & F University, Yangling 712100, China; Key Laboratory of Livestock Biology, Northwest A&F University, Yangling 712100, China; College of Veterinary Medicine, Northwest A&F University, Yangling 712100, China; Shaanxi Centre of Stem Cells Engineering & Technology, Northwest A & F University, Yangling 712100, China; Key Laboratory of Livestock Biology, Northwest A&F University, Yangling 712100, China; Key Laboratory of Livestock Biology, Northwest A&F University, Yangling 712100, China; Department of Animal Biotechnology, College of Veterinary Medicine, Northwest A&F University, Yangling 712100, China; BoYu Intelligent Health Innovation Laboratory, Hangzhou 311121, China; Key Laboratory of Organ Regeneration and Reconstruction, State Key Laboratory of Stem Cell and Reproductive Biology, Institute of Zoology, Chinese Academy of Sciences, Beijing 100101, China; State Key Laboratory of Animal Biotech Breeding, College of Biological Sciences, China Agricultural University, Beijing 100193, China; RNA Biomedical Institute, Sun Yat-Sen Memorial Hospital, Zhongshan School of Medicine, Sun Yat-Sen University, Guangzhou 510080, China; College of Veterinary Medicine, Northwest A&F University, Yangling 712100, China; Shaanxi Centre of Stem Cells Engineering & Technology, Northwest A & F University, Yangling 712100, China; Key Laboratory of Livestock Biology, Northwest A&F University, Yangling 712100, China; Northwest A&F University Shenzhen Research Institute, Shenzhen 518057, China

**Keywords:** DUX, phase separation, totipotency-like state, MERVL, super-enhancers, 3D genome reorganization

## Abstract

The acquisition of totipotency requires transcriptional activation of endogenous retroviruses (MERVL/HERVL) and zygotic genome activation (ZGA) related genes, yet the molecular mechanisms linking chromatin architecture to this process remain elusive. Here, we demonstrate that mouse Dux and human DUX4, double homeobox transcription factors essential for totipotency, form liquid–liquid phase-separated (LLPS) condensates through conserved arginine residues within intrinsically disordered regions (IDRs) in the Homeobox domain. These condensates recruit CBP/p300 and CTCF to establish super-enhancers (SEs) at MERVL/MT2 loci, enabling H3K27ac deposition and chromatin accessibility. Hi-C analysis revealed that DUX-driven phase separation facilitates 3D genome reorganization, including *de novo* formation of enhancer-promoter loops and TAD boundary shifts. Disruption of LLPS (DUX^R70A^) abolished SE assembly, transcriptional activation, and embryonic chimerism. Strikingly, human DUX4 required phase separation for both myotoxic gene activation and cytotoxicity in facioscapulohumeral muscular dystrophy (FSHD) models. Our study establishes a paradigm wherein phase separation integrates transcriptional control with 3D genome remodeling to license totipotency, with direct implications for developmental biology and disease therapy.

## Introduction

Liquid–liquid phase separation (LLPS) exerts complicated regulatory control over a spectrum of biological processes (Liu et al., 2025). In embryonic stem cells (ESCs), pluripotency factors such as OCT4 leverage intrinsically disordered regions (IDRs) to form phase-separated condensates at super-enhancers (SEs), which orchestrate cell identity by activating lineage-specific genes (Boija et al., 2018; Sabari et al., 2018). While LLPS-mediated transcriptional control is well-documented in pluripotency, its role in totipotency—the capacity of a single cell to generate all embryonic and extraembryonic lineages—remains poorly understood. Recent advances in capturing totipotent-like cells *in vitro* (Hendrickson et al., 2017; Hu et al., 2020, 2023; Iturbide et al., 2021; Li et al., 2024; Macfarlan et al., 2012; Mazid et al., 2022; Shen et al., 2021; Taubenschmid-Stowers et al., 2022; Zhang et al., 2019) have provided models to dissect this elusive developmental state. However, the molecular mechanisms linking chromatin architecture, transcriptional activation of endogenous retroviruses (e.g., MERVL/HERVL), and zygotic genome activation (ZGA) to totipotency remain unresolved.

The double homeodomain transcription factor DUX is a master regulator of totipotency-like in mice, driving the expression of two-cell (2C)-specific genes and retrotransposons (De Iaco et al., 2017; Eckersley-Maslin et al., 2019; Grow et al., 2021; Hendrickson et al., 2017; Whiddon et al., 2017). Despite its pivotal role, only a subset of DUX-activated genes directly bind to its target motifs (Iturbide and Torres-Padilla, 2017), suggesting an indirect regulatory mechanism involving chromatin reorganization. Intriguingly, totipotency-like cells exhibit extensive chromatin accessibility and H3K27ac deposition at retrotransposon-rich loci (Hendrickson et al., 2017), yet how DUX coordinates these epigenetic and architectural changes remains unclear. While studies implicate coactivators like CBP/p300 in DUX4-mediated H3K27 acetylation (Choi et al., 2016), the spatial and dynamic regulation of these interactions has not been explored. Furthermore, whether phase separation underpins DUX’s ability to remodel the 3D genome and establish totipotency-like-specific SEs remains unknown.

Here, we investigate how DUX-family proteins utilize phase separation to integrate transcriptional activation with 3D genome reorganization during the transition from pluripotency to totipotency-like. We demonstrate that mouse Dux and human DUX4 form phase separation-driven condensates via conserved arginine residues within their IDRs, enabling recruitment of CBP/p300 and CTCF to retrotransposon-associated SEs. Through Hi-C and functional assays, we reveal that DUX condensates reconfigure enhancer-promoter loops and TAD boundaries, licensing transcriptional activation of MERVL/2C genes. Disruption of phase separation abolishes SE assembly, retrotransposon activation, and embryonic chimerism, underscoring its necessity for totipotency. Strikingly, we extend these findings to facioscapulohumeral muscular dystrophy (FSHD), where phase separation-deficient DUX4 mutants mitigate cytotoxicity, highlighting therapeutic potential. Our work establishes a paradigm wherein phase separation bridges transcriptional control and 3D genome remodeling to unlock totipotency, offering insights into developmental biology and disease mechanisms.

## Results

### DUX serves as a conserved master regulator of totipotency across species and cellular models

**Figure 1.**
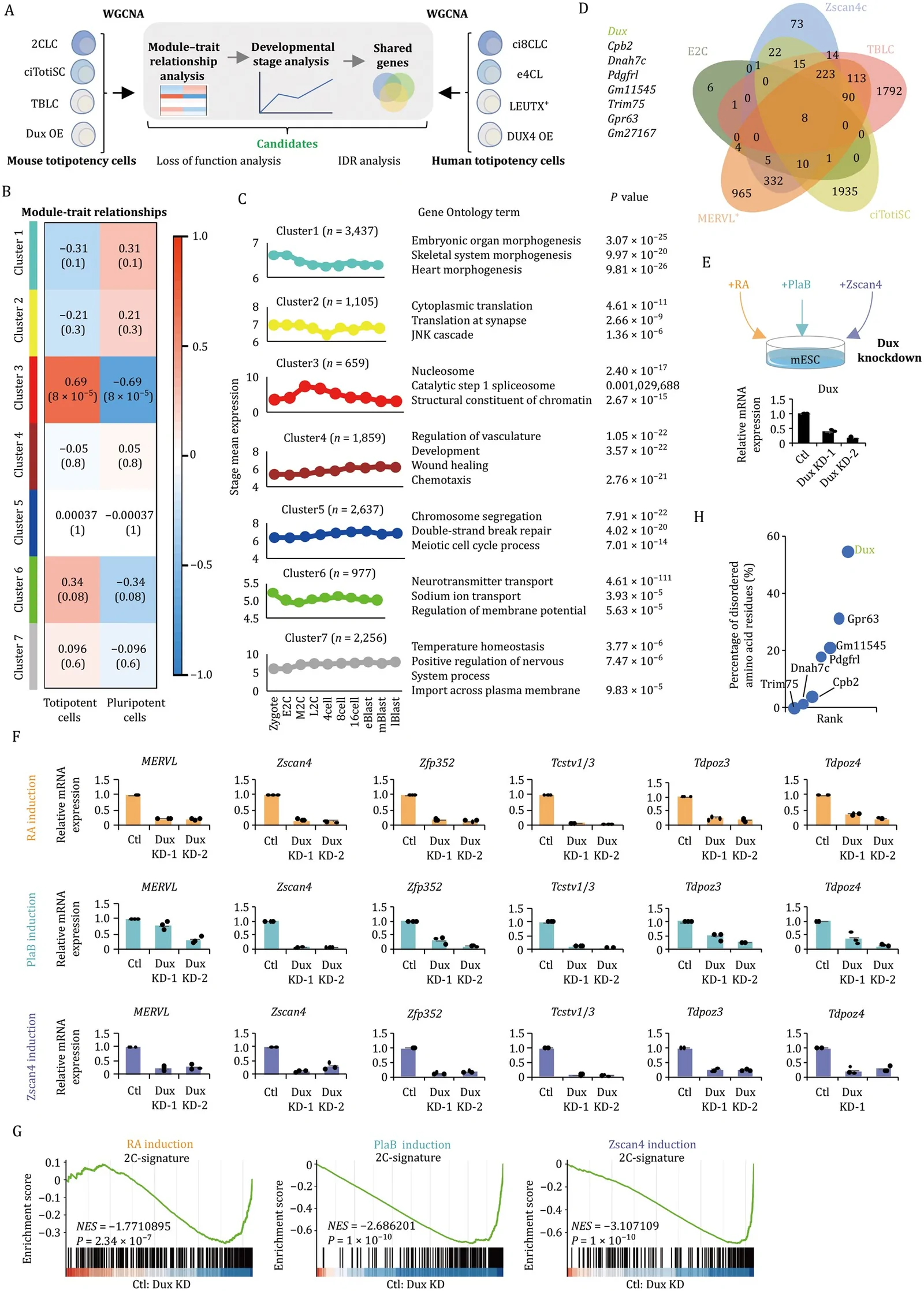
**DUX is essential for totipotent cells with different origins**. (A) Flow chart for screening key transcription factors in mouse and human totipotency cells. (B) Module–trait relationship analysis. The values within each cell represent the correlation coefficient of the module—eigen with sample type (trait). The corresponding *P*-values for each module are provided in parentheses. (C) Seven clusters of genes exhibit stage-specific expression patterns during mouse pre-implantation development. Representative enriched Gene Ontology terms along with their corresponding *P-*values are presented on the right. (D) Venn diagram illustrating those eight genes from cluster E2C was concurrently upregulated in multiple models of totipotent cells, including MERVL^+^, Zscan4c overexpression, TBLC, and ciTotiSC. (E) Knockdown of Dux in RA-induced, PlaB-induced and Zscan4-induced totipotent cells. (F) Relative expression levels of *MERVL*/2C genes in RA-induced, PlaB-induced, and Zscan4-induced totipotent cells following Dux knockdown were assessed RT-qPCR data are presented as average ± SD. (G) GSEA demonstrates concordant suppression of totipotency programs upon Dux knockdown across different induction models of Fig. 1E. (H) IDR analysis of eight genes in (D). The IDR% values were obtained using the MobiDB database (mobidb.org/) (Piovesan et al., 2021), which integrates curated structural disorder data from PDB and DisProt with consensus results from multiple disorder prediction algorithms.

Recently, totipotent cells with expanded development potential have been established by using chemical reprogramming or gene overexpression. To systematically identify core drivers of totipotency, we performed weighted gene co-expression network analysis (WGCNA) (Huang et al., 2014) on transcriptomes from diverse totipotent cells, including chemically reprogrammed, gene-overexpressing, and retrotransposon-activated models, alongside pluripotent controls (Fig. 1A and Table S1) (Hendrickson et al., 2017; Hu et al., 2023; Iturbide et al., 2021; Shen et al., 2021; Zhao et al., 2018). Among seven co-expression modules, a single module (Red) exhibited exclusive correlation with the totipotent state (Figs. 1B, S1A and S1B). Genes within this module were enriched for transcriptional regulation and chromatin-associated processes, including “structural constituent of chromatin” and “spliceosome” (Fig. 1C), mirroring molecular events during early embryogenesis (Malik and Wang, 2022). Temporal clustering revealed that 65 zygote/early two-cell (E2C)-activated genes formed the core of this module (Fig. S1C), with eight genes—*Dux*, *Cpb2*, *Dnah7c*, *Pdgfrl*, *Gm11545*, *Trim75*, *Gpr63*, and *Gm27167*—sharing conserved upregulation across all totipotent models (Fig. 1D).

Next, we sought to investigate whether the regulators of mouse totipotency are conserved in humans. Using the strategy outlined in Fig. 1A, we identified gene networks in human totipotent cells (Fig. S1D–G) (Hendrickson et al., 2017; Li et al., 2024; Mazid et al., 2022; Zhang et al., 2025). Interspecies analysis identified 68 human orthologs within totipotency-related networks, with DUX4 (including isoforms DUXA and DUXC) representing the sole overlap with mouse regulators (Fig. S1H). Subsequently, we knocked down Dux using specific shRNAs previously reported (Yang et al., 2020), and observed that the depletion of Dux impaired the activation of MERVL/2C genes in RA-induced (Iturbide et al., 2021), PlaB-induced (Shen et al., 2021), and Zscan4-induced (Zhao et al., 2018) totipotent cells (Fig. 1E–G). In conclusion, building upon previous studies that have demonstrated a role for Dux in totipotent conversion (Hendrickson et al., 2017; Whiddon et al., 2017), we provide a systematic analysis revealing Dux as a common regulatory factor across different totipotent-like cell models.

### IDR-driven DUX phase separation licenses totipotent transcriptional condensate formation

**Figure 2.**
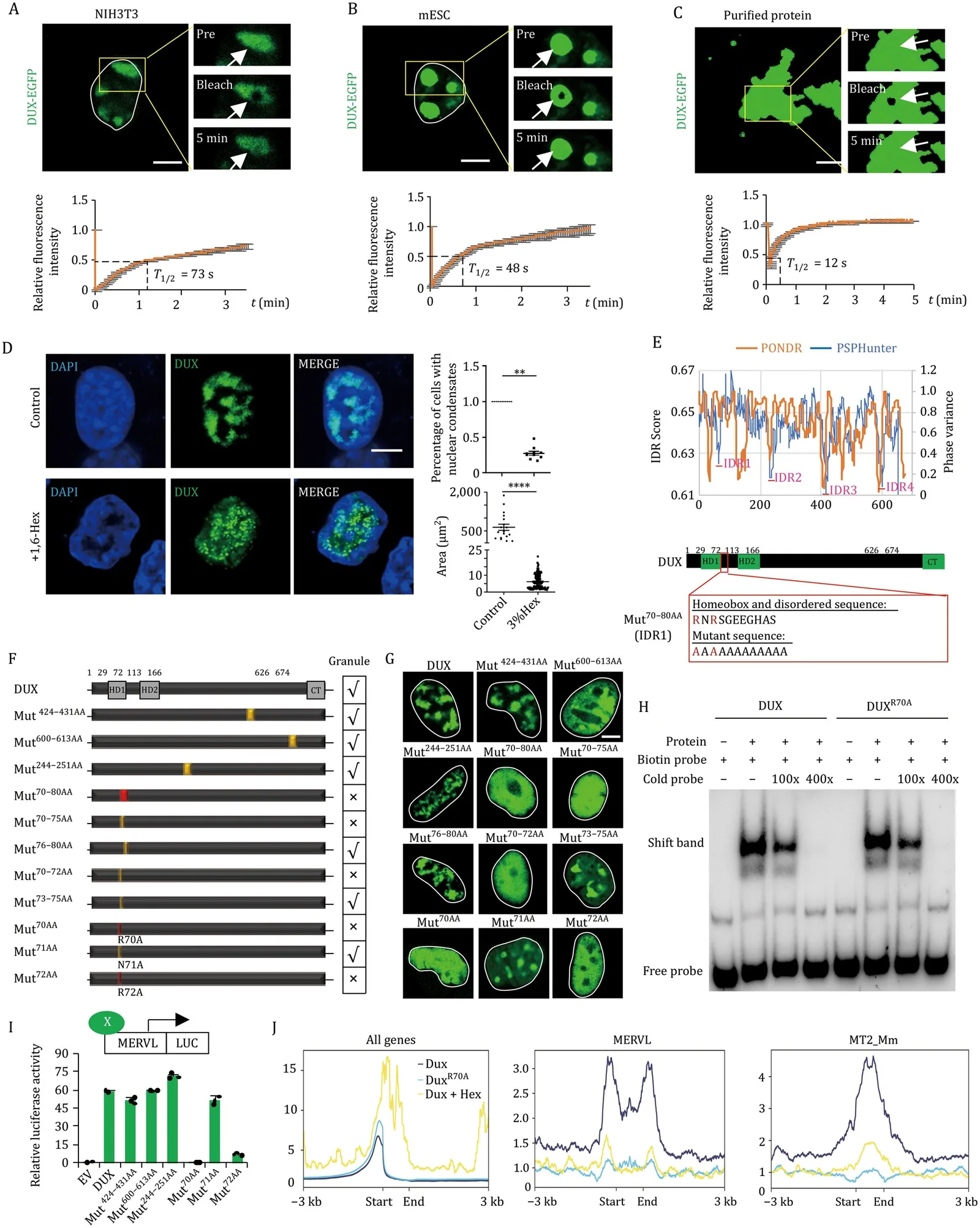
**DUX undergoes condensates *in vitro* and *in vivo***. (A and B) Fluorescence recovery after photobleaching (FRAP) of EGFP-DUX condensate in NIH3T3 (A) (*n* =10 cells) and mESCs (B) (*n* =7 cells) bleached at positions indicated by white arrows. Quantification of fluorescence intensity during FRAP assay (bottom). Scale bars: 5 μm. (C) FRAP of EGFP-DUX purified protein condensation *in vitro* (*n* = 6 condensates). Quantification of fluorescence intensity during FRAP assay (bottom). (D) Live-cell fluorescence imaging revealed that DUX condensates were disrupted by treatment with 1,6-hexanediol (1,6-Hex; 3%) (left panel). The analysis of condensate number (upper right panel) and condensate area (lower right panel) following 1,6-Hex treatment was conducted using three biological replicates. Data are represented as the mean ± SEM. Statistical significance was determined by *t*-test: **P < 0.01; ****P < 0.0001. Scale bars: 5 μm. (E) Graphs depicting the predicted intrinsic disorder regions and driving residues of DUX as determined by PONDR (pondr.com) and PSPHunter (psphunter.stemcellding.org/index.php). (F) Overview of the DUX mutants utilized to identify specific domains or amino acid residues responsible for phase separation. The symbols “√” and “×” denote the capability and incapability of phase separation, respectively. (G) Live-cell images revealed the localization of EGFP-tagged DUX mutants (in Fig. 2G) within transfected cells. Scale bars: 5 μm. (H) EMSA showed the DNA-binding capability of DUX and DUX^R70A^ in the presence or absence of cold probe. The probe sequence is composed of the motif of DUX located on MERVL (Whiddon et al., 2017). (I) Dual luciferase assay to detect core region of MERVL activation induced by various DUX. (J) Occupancy profile of DUX in CUT&Tag peaks averaged over all genomic regions (left), MERVL (middle), or MT2_mM (right) under different conditions.

In recent years, a growing body of evidence has suggested that many transcription regulators in pluripotent cells function through liquid–liquid phase separation (LLPS). However, this emerging paradigm has not yet been fully established in the field of totipotent cells, as there are no reports indicating that key regulators of totipotency have undergone phase separation. To investigate whether totipotency regulators can form biomolecular condensates, we overexpressed the DUX fused with EGFP in cellular models. Notably, the DUX-EGFP fusion protein was observed to form nuclear biomolecular condensates. Following a comparison with previous studies (Grow et al., 2021; Hendrickson et al., 2017; Vega-Sendino et al., 2024; Xie et al., 2022), we further confirmed the formation of DUX protein foci in cells. Additionally, we conducted fluorescence recovery after photobleaching (FRAP) analysis to examine the dynamics of DUX protein condensation both *in vivo* (Fig. 2A and 2B) and *in vitro* (Fig. 2C). Although the size and morphology of DUX-EGFP condensates differ slightly between NIH3T3 and ESC cell lines, the GFP signal of the DUX-EGFP fusion protein in both cell types rapidly recovers after photobleaching. This suggests that DUX condensates are highly dynamic and freely exchanges with cellular components under physiological conditions. To investigate whether these condensates exhibit liquid demixing properties, we treated them with 1,6-hexanediol, a reagent known to disrupt liquid-like condensates (Kroschwald et al., 2017). The results showed that the condensates were indeed disrupted (Fig. 2D).

To elucidate the mechanisms underlying the formation of these condensates, we first analyzed the primary sequence of DUX using the PLAAC algorithm, which assesses amino acid compositional similarity to prion-like domains (PrLDs) (Lancaster et al., 2014). This analysis identified two regions within DUX that may constitute PrLDs (Fig. S2A). However, the removal of these PrLDs had no effect on DUX condensation (Fig. S2B). Recent studies have shown that weak promiscuous interactions caused by IDRs can drive protein condensation (Kim et al., 2023). We found that a significant portion of the DUX protein contains IDRs proposed to LLPS (Fig. 2E). This finding is consistent with DUX having the highest proportion of IDRs among the identified proteins (Fig. 1H). To pinpoint the molecular determinants of phase separation, we systematically truncated IDR segments (Fig. S2C). Deletion of the IDR spanning residues 70–80 (IDR1^Mut^, hereafter referred to as Dux^Mut^) abolished condensate formation (Fig. S2D), while mutagenesis of conserved arginine residues (R70A/R72A) recapitulated this defect *in vivo* and *in vitro* (Figs. 2F, 2G, S2E, and S2F). Purified wild-type DUX formed liquid-like condensates under physiological conditions, whereas DUX^R70A^ failed to phase-separate even at high concentrations (Fig. S2E and S2F), establishing arginine-mediated weak interactions as critical for phase separation.

Functional relevance was confirmed using a MERVL-driven luciferase reporter. Accordingly, we first identified the core sequence of MERVL targeted by DUX (Fig. S2G and S2H) and subsequently evaluated the transcriptional activation activity of various DUX mutants using a dual-luciferase assay. While wild-type DUX robustly activated MERVL transcription, phase separation-deficient mutants (DUX^R70A^, DUX^R72A^) lost this capacity (Fig. 2I), despite electrophoretic mobility shift assays demonstrating that DUX^R70A^ retained the ability to bind the MERVL element (Fig. 2H). Consistent with our live-cell imaging recordings, DUX condensates gradually formed upon Dux expression, followed by the activation of MERVL approximately one hour later (Videos S1–S3). CUT&Tag profiling further revealed that, although 1,6-hexanediol treatment modestly increased genome-wide DUX occupancy, the reduction was far more pronounced at MERVL/MT2 elements, similar to the pattern observed for phase separation-deficient mutants (Fig. 2J). Intriguingly, adding MERVL (125–375) DNA fragments enhanced DUX condensation *in vitro* (Fig. S2I and S2J), suggesting a feedback loop where DNA binding stabilizes phase-separated hubs. These findings indicate that the ability of DUX to form phase-separated condensates is critical for its efficient recruitment to totipotency-associated genomic loci enriched in MERVL/MT2_Mm elements.

### IDR-mediated DUX condensates drive the formation of totipotency-specific super-enhancers

**Figure 3.**
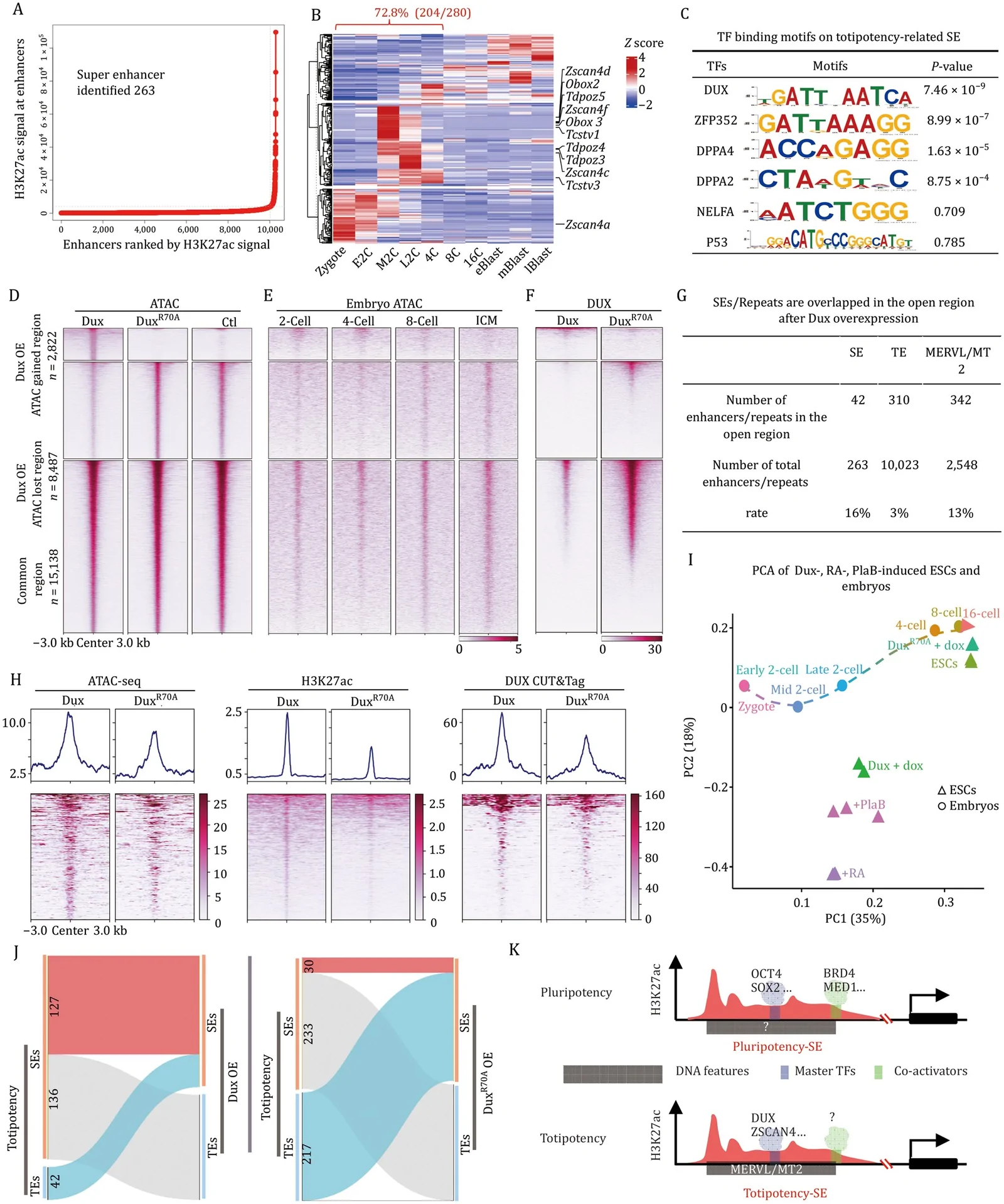
**Phase-separation of DUX activates MERVL/2C genes through mediating super-enhancers establishment**. (A) Identification of SEs in 2CLCs (MERVL^+^). Within the 12.5-kb window, H3K27ac signals were ranked for 2CLCs. The enhancers above the inflection point were defined as totipotent-related SEs. (B) Expression changes of totipotency-associated genes within SEs (*n* = 335) from the mouse zygote to the blastocyst stage. (C) Enrichment analysis of TF binding motifs in 2CLCs using the simple enrichment analysis (SEA) algorithm from MEME. Motifs of reported totipotency-related TFs were analyzed in the SE sequences. (D-F) Heat maps illustrate regions of ATAC-seq signal gain, loss, and common peaks in Control (Ctl), Dux-overexpressing (Dux OE), and phase separation-deficient Dux^R70A^ samples. Dux OE cells exhibit an open/closed chromatin landscape that resembles that of the two-cell-stage embryo. CUT&Tag signals for Dux are enriched in regions of ATAC-seq signal gain, whereas Dux^R70A^ is associated with regions of ATAC-seq signal loss. (G) Ratio of Dux gain regions containing SEs, TEs, or MERVL/MT2. (H) Heatmaps showing ATAC-seq, H3K27ac, and DUX signal profiles of totipotency-related SEs loci after Dux and phase separation deficiency Dux^R70A^ overexpression (*n* = 2 biological replicates). (I) Transcriptome-based PCA of ESCs, induced cells and mouse preimplantation embryos. (J) Sankey diagrams for totipotency SEs change after overexpressing Dux and phase separation deficient Dux^R70A^. The red bars indicate the 263 reference SEs in 2CLCs, allowing direct comparison across conditions. Red blocks indicate SEs that are maintained across both conditions. Blue blocks represent regions originally identified as TEs in 2CLCs that are converted to SEs under Dux OE or Dux^R70A^ OE. Grey blocks represent regions classified as SEs in 2CLCs that are downgraded to TEs under Dux OE or Dux^R70A^ OE. (K) Models of the characteristics of pluripotent SE and totipo tency SE.

Super-enhancers play a pivotal role in determining cell identity by modulating cell-type-specific genes (Huang et al., 2019). The mechanisms by which DUX regulate enhancer-dependent transcription programs during the pluripotency-to-totipotency (P-T) transition remain poorly understood. To elucidate how DUX influences super-enhancer assembly during the transition to totipotency, we aimed to identify super-enhancers in totipotency-like cells by mapping H3K27ac-marked enhancers in MERVL-positive two-cell-like cells (2CLCs) (Zhu et al., 2021). Using the ROSE algorithm, we identified 263 SEs and 10,022 typical enhancers (TEs) in the 2CLCs (Fig. 3A). Notably, SEs were significantly longer (17-fold) and more frequently associated with MERVL/MT2 elements (26.2%) compared to TEs (7.9%) (Fig. S3A and S3C). These SEs were enriched near intergenic and promoter regions (Fig. S3B). Strikingly, approximately 72.8% of SE-associated genes were activated before the four-cell embryo stage, compared to 27.2% activated at later stages (Fig. 3B; e.g., Tdpoz3/4 and Gm49303/2022; Fig. S3D), suggesting a functional connection between retrotransposon-derived SEs and totipotency.

SEs are known to cluster transcription factor binding motifs (Whyte et al., 2013). Motif analysis revealed that SEs were enriched for binding sites of totipotency-related transcription factors, including DUX, ZFP352, and DPPA2/4 (Fig. 3C), many of which have been shown to promote the P-T transition (Eckersley-Maslin et al., 2019; Li et al., 2023, Whiddon et al., 2017). DUX exhibited the strongest correlation with H3K27ac deposition (Fig. S3E), indicating potential interaction with coactivators to catalyze histone acetylation. ATAC-seq analysis demonstrated that DUX overexpression increased chromatin accessibility at 2,822 genomic regions, many of which were enriched in two-cell embryos (Fig. 3D and 3E). Strikingly, these regions lost accessibility upon expression of the phase separation-deficient Dux^R70A^ mutant (Fig. 3D and 3E) as well as enrichment of this mutant (Fig. 3F), underscoring the necessity of phase separation for chromatin remodeling. To investigate whether DUX condensate-induced chromatin remodeling is essential for SE assembly, we examined DUX-induced open chromatin regions, which were found to contain 13% MERVL/MT2 elements (Fig. 3G). Given that totipotency-like-associated SEs also harbor MERVL/MT2, we analyzed these SEs and found that 16% of them (vs. 3% of TEs) coincided with DUX-induced open regions (Fig. 3G). These results indicate that DUX-mediated phase separation is critical for chromatin remodeling during totipotency-like SE assembly.

To further assess the direct impact of DUX phase separation on SE assembly as well as targeted genes, we analyzed ATAC-seq signals in Dux^R70A^-expressing cells. SE-associated open chromatin regions exhibited reduced accessibility (Fig. 3H, left), accompanied by diminished H3K27ac deposition and phase separation-deficiency DUX enrichment (Fig. 3H, middle and right). The reduction in chromatin accessibility at SEs, mediated by the phase separation-dependent function of DUX, accompanies the transcriptional silencing of adjacent two-cell (2C) genes, such as *Obox* and *Tdpoz* (Fig. S3F). Consequently, Dux^R70A^ expressing cells displayed a transcriptomic shift from a totipotency-like to a pluripotent stem cell profile (Figs. 3I, S3G, and S3H). The number of totipotency-like-associated SEs also decreased significantly (11% vs. 48% in DUX-overexpressing cells; Fig. 3J), further highlighting the role of phase separation in SE assembly.

Collectively, we initially identified totipotency-like-associated SEs that contain the retroviral element MERVL specifically targeted by totipotency-related proteins, which are distinctly different from pluripotency-related SE. Additionally, chromatin remodeling induced by phase separation of DUX will facilitate SE assemble for the activation of totipotency-related transcriptome. However, the coactivators involved in the establishment of these totipotency-like SE remain unclear (Fig. 3K).

### DUX condensates spatially reorganize CBP/p300 to catalyze totipotency-specific epigenetic remodeling

**Figure 4.**
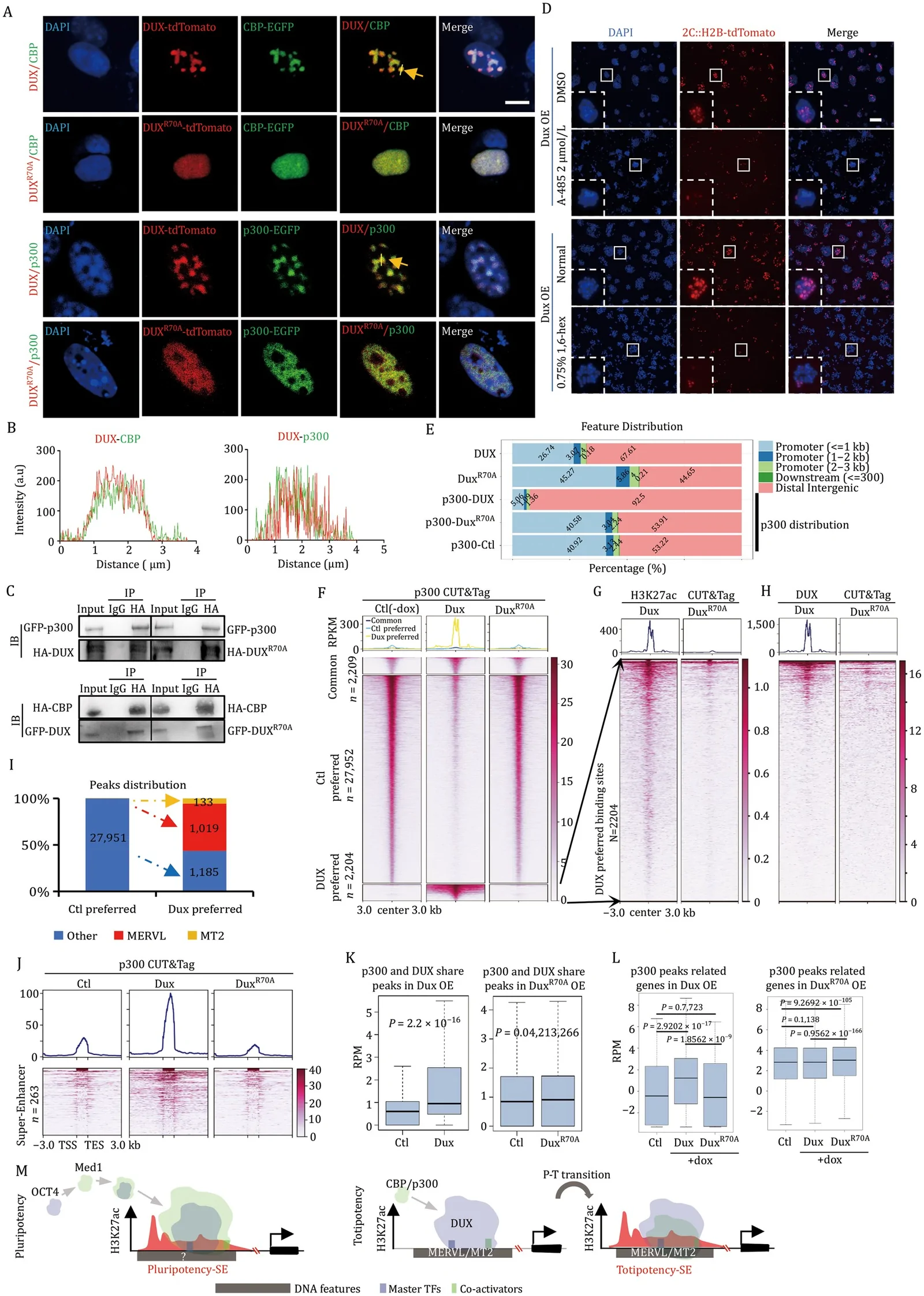
**Dux recruits factors to catalyze acetylation within totipotency-related super-enhancer**. (A) Fluorescence images of the cell expressing tdTomato-Dux or tdTomato-Dux^R70A^ co-localized with EGFP-p300 and EGFP-CBP in the same cell. Scale bars: 5 µm. (B) Quantification of the fluorescence intensity along the line profile embedded within the condensate indicated by the arrow in Fig. 4A. (C) Co-IP analysis of GFP-p300 with HA-DUX/DUX^R70A^, and HA-CBP with GFP-DUX/DUX^R70A^. (D) Fluorescence microscopy images illustrating the alterations in MERVL-positive cell populations following Dux induction, after treatment with either A-485 or 1,6-Hex (n = 3 biological replicates). Scale bars: 50 µm. (E) Stacked plots illustrating the changes in the distribution percentages of Dux and p300 peaks following the overexpression of Dux or the phase separation-deficient mutant Dux^R70A^. (F) Heatmap illustrating enrichment of p300 in control, Dux-induced, and DUX^R70A^-induced cells. Following Dux expression, a Dux-preferred region of p300 enrichment was identified, comprising 2,204 peaks. After overexpression of Dux and phase separation-deficient Dux^R70A^, three distinct regions were defined: Dux-preferred, control-preferred, and common regions. By comparing the differential peaks of p300 between control and Dux + dox conditions, these regions were categorized accordingly. (G and H) Heatmap illustrating the enrichment of H3K27ac (G) and Dux (H) in the Dux-preferred region of p300 following overexpression of Dux and phase separation deficiency Dux^R70A^. (I) Stacked plots showing the distribution of Ctl- and Dux-preferred p300 binding peaks identified in (F). (J) Heatmap showing enrichment of p300 on totipotency-related SEs (number = 263) after overexpression of Dux and phase separation deficiency Dux^R70A^. (K) Box plots illustrating the expression levels (in reads per million, RPM) of loci commonly shared by p300 and Dux/Dux^R70A^ in the corresponding samples. (L) Box plots illustrating the expression levels (in reads per million, RPM) of neighboring genes correlated with loci co-occupied by p300 and Dux/Dux^R70A^ in the respective samples. (M) Model of the establishment mechanism of pluripotent SE and totipotency SE.

Chromatin architecture can be modified through various post-translational modifications of histone proteins, thereby relaxing the tightly packed chromatin structure (Perino and Veenstra, 2016). H3K27ac serves as a marker for active enhancers and plays a role in this process. However, the mechanism by which DUX regulates H3K27ac modification to establish totipotency-like-associated SE in regions where chromatin remains closed in pluripotent cells is not fully understood. We investigated how the phase separation of DUX activates H3K27ac and observed that DUX condensates colocalized with H3K27ac (Fig. S4A and S4B). Additionally, overexpression of DUX directly increased both H3K27ac and H3K4me1 levels in cells (Fig. S4C and S4D). The recruitment of histone acetyltransferases CBP/p300 is critical for establishing super-enhancers (SEs) marked by H3K27ac (Sen et al., 2019). We investigated whether DUX-driven phase separation spatially directs CBP/p300 activity to retrotransposon-rich loci during the P-T transition. Live-cell imaging revealed that DUX condensates colocalized with both CBP and p300 (Fig. 4A and 4B), whereas the phase separation-deficient mutant Dux^R70A^ failed to form condensates with the coactivators (Figs. 4A and S4E). Notably, CBP and p300 colocalize with DUX in cells expressing either wild-type DUX or phase separation-deficient DUX^R70A^. Co-immunoprecipitation (Co-IP) confirmed that DUX physically interacts with CBP/p300 independently of phase separation (Fig. 4C), suggesting that phase separation spatially concentrates, rather than initiates, these interactions.

To investigate whether the condensates formed by DUX and its interaction with CBP/p300 are essential for DUX-induced totipotency, we treated DUX-expressing cells with the CBP/p300 inhibitor A-485 or shRNA-specific for CBP and p300. These treatments resulted in the abolition of MERVL reporter activity (Figs. 4D, S4F, and S4G) and disrupted MERVL/2C gene expression (Fig. S4H), findings consistent with observations in cells where DUX condensates were disrupted by 1,6-hexanediol. Conversely, co-expression of DUX with p300 synergistically enhanced MERVL activation (Fig. S4I), further implicating phase separation induced interaction with CBP/p300 is essential for DUX function. Notably, the concentration of 1,6-hexanediol used in this experiment (0.75%) was relatively low and significantly below the levels typically used to disrupt nucleoli or non-nucleolar compartments (Liu et al., 2021; Vertii et al., 2019; Xie et al., 2022), supporting the specificity of our observations.

Considering that CBP/p300 is recruited to the condensates formed by DUX, we investigated whether phase separation of DUX affects p300 distribution. Indeed, DUX condensates reprogram the genome-wide binding of p300 towards a DUX-binding pattern. Upon DUX overexpression, p300 preferentially binds to intergenic regions (92.5% versus 53.91% in the control and Dux^R70A^ groups), which is consistent with the altered DUX enrichment following the loss of R70 (67.61% versus 44.65%) (Fig. 4E). To elucidate the binding characteristics of p300 influenced by DUX, we profiled genome-wide p300 binding in DUX-overexpressing cells and identified 2,204 “DUX-preferred” binding regions (Fig. 4F). These regions exhibited elevated H3K27ac and DUX occupancy (Fig. 4G and 4H). Strikingly, these regions contain more acetylated MERVL and MT2 (Figs. 4I and S4J), consistent with p300's preferential binding to SEs in DUX-expressing cells (Fig. 4J). Through Dux^R70A^ expression maintains some loci along with p300 enrichment (Fig. 4H), phase separation deficiency fails to reactivate loci for enhancer RNA production (Figs. 4K and S4K) or activate nearby gene expression with comparable intensity to that of the wild type (Figs. 4L and S4K). This demonstrates that phase separation licenses “spatially restricted” coactivator recruitment for gene activation.

Mechanistically, DUX condensates create microenvironments where p300/CBP catalyzes H3K27ac deposition, rendering chromatin accessible for transcriptional machinery (Fig. 4M). This contrasts with pluripotency, where pre-existing SEs recruit OCT4 and coactivators to stabilize lineage-specific enhancers (Sabari et al., 2018). Our findings redefine the role of DUX as an architectural scaffold that phase separates to spatially redistribute epigenetic modifiers, enabling retrotransposon-driven SE establishment—a hallmark of totipotency.

### DUX condensates drive enhancer-promoter interactions critical for totipotency

**Figure 5.**
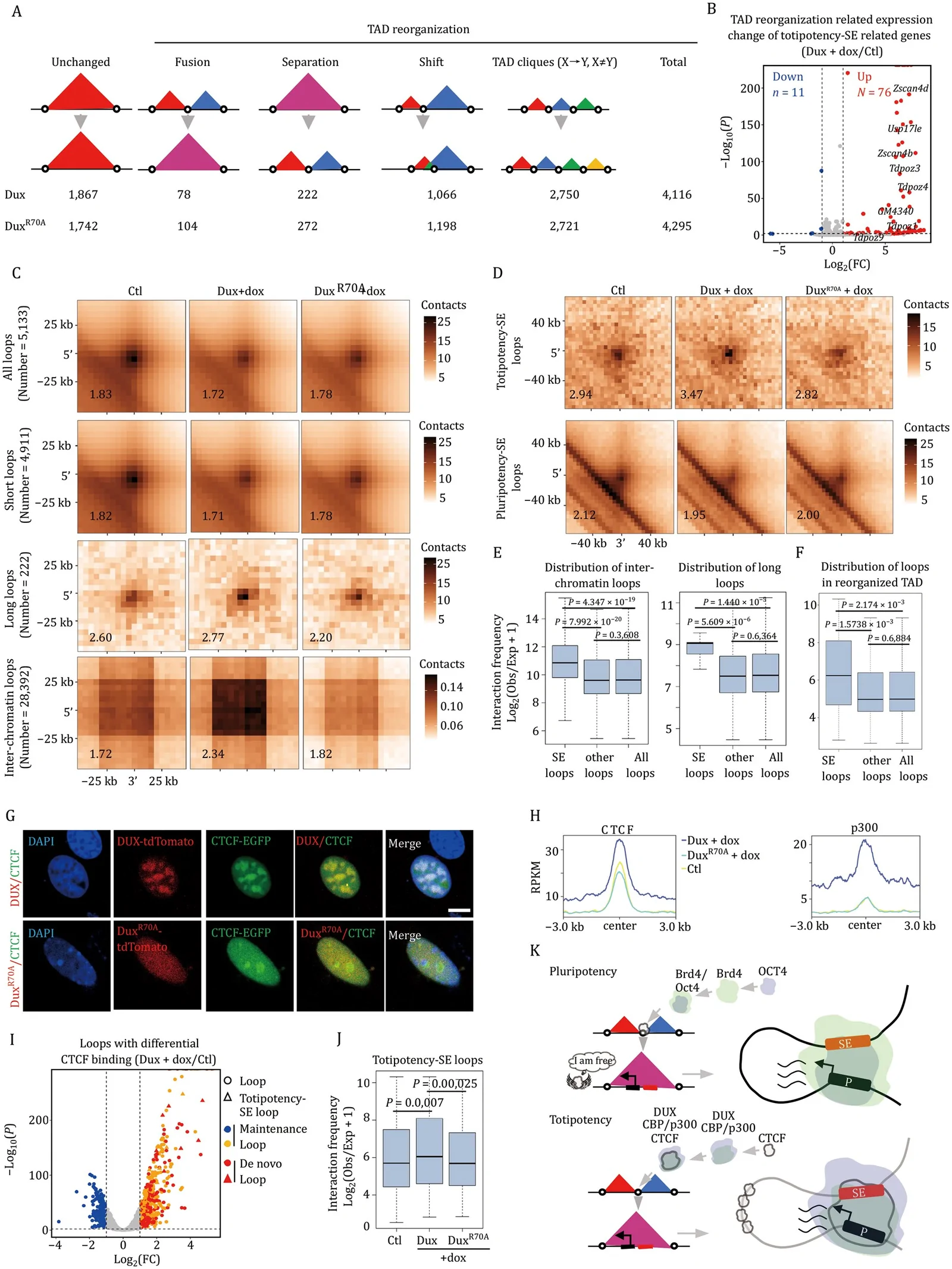
**DUX condensates induce totipotency-related enhancer-promoter interaction**. (A) Comparison of the number of unchanged TADs versus reorganized TADs following the overexpression of Dux and DuxR70A, respectively. TAD reorganization includes TAD fusion, separation, shift, and TAD cliques (X, Y > 2). (B) Volcano plot illustrating the expression changes of totipotency-SE associated genes related to TAD reorganization in cells overexpressing Dux. Gray dots represent genes with no significant change in expression; red dots indicate upregulated genes; blue dots signify downregulated genes. The majority of SE-associated genes exhibited upregulation following TAD reorganization in Dux-overexpressing cells (right panel), as opposed to those overexpressing DUX^R70A^. (C) Aggregate peak analysis (APA) was employed to quantify the strength of all loops, including short loops (middle), long loops (middle), and inter-chromatin loops (bottom) following the overexpression of Dux and phase separation deficiency Dux^R70A^. The mean APA enrichment scores are presented in the lower left APA plot. Short loops are defined as chromatin loops with a genomic span less than 1,000 kb, whereas long loops are those spanning more than 1,000 kb. Inter-chromatin loops refer to loops formed between loci located on different chromosomes (i.e., inter-chromosomal interactions). (D) APA was employed to quantify the totipotency-SE loops and pluripotency-SE loops (bottom) after overexpression of Dux and phase separation deficiency Dux^R70A^. The mean APA enrichment scores were shown in the lower left APA plot. (E) Distribution of inter-chromatin loops (left) or long loops (right) for SE loops, other loops and all loops. (F) Distribution of loops in reorganized TAD for SE loops, other loops, and all loops. (G) Fluorescence microscopy images demonstrate co-localization of tdTomato-Dux and tdTomato-Dux^R70A^ with EGFP-CTCF within the same cellular environment (*n* = 3 biological replicates). Scale bars: 5 µm. (H) Enrichment of CTCF and p300 in CTCF-up loops. (I) Volcano plot comparing differential CTCF binding loops in Dux-expressing cells against control (Ctl). Gray dots represent loops with no significant change in CTCF binding; blue dots indicate loops where CTCF binding is weakened; red dots signify loop where CTCF binding is strengthened; orange dots highlight newly generated loops with strengthened CTCF binding; triangles denote loops associated with SE. (J) Quantification of interaction frequency of totipotency-SE loops, detected in Ctl, Dux overexpressing, and Dux^R70A^ overexpressing ESCs. (K) Model of the establishment mechanism for pluripotent-related DNA loops/TAD and totipotency-related DNA loops/TAD.

Super-enhancers (SEs) regulate cell identity by orchestrating chromatin architecture and enhancer-promoter communication (Huang et al., 2019). While phase separation has been implicated in pluripotency-associated SE regulation (Sabari et al., 2018), its role in establishing SE-specific chromatin loops remain unexplored. To investigate this, we generated Hi-C profiles of mESCs after overexpressing Dux or Dux^R70A^, examining Dux’s ability to modulate 3D chromatin structure via phase separation. Hi-C analysis of Dux-overexpressing embryonic stem cells (ESCs) revealed widespread chromatin reorganization, with 70.5% (4226/5992) of genomic regions undergoing TAD boundary shifts, fusions, or *de novo* formations (Figs. 5A and S5A). These reorganized TADs were enriched for totipotency-like-related SEs with (75.7%, 199/263) or without DUX condensates (65.8%, 173/263) (Fig. S5B), while only genes within DUX condensates induced reorganized TADs exhibited pronounced upregulation (Figs. 5B and S5C). Strikingly, Dux condensates facilitated long-range (>1 Mb) and inter-chromosomal interactions, as evidenced by elevated contact frequencies in Hi-C aggregate peak analysis (APA) (Fig. 5C). SE-associated loops also showed enhanced interaction strength in Dux-expressing cells, whereas pluripotency-associated SE loops remained unaffected (Fig. 5D). These totipotency-like-associated SE loops preferentially localize within inter-chromatin (Fig. 5E, right), long-range chromatin regions (Fig. 5E, left) and reorganized TADs regions (Fig. 5F). These findings suggest that Dux condensates selectively reconfigure chromatin topology to prioritize totipotency gene activation.

CTCF, a key architectural protein, was dynamically recruited to Dux condensates (Fig. 5G) thereby impacting genome-wide CTCF binding. In fact, distinct binding motif of totipotent regulatory factors, including DUX and p53, was observed within DNA loops in Dux-expressing cells compared to Dux^R70A^ and control conditions (Fig. S5D), suggesting a potential collaborative role of CTCF with DUX in the establishment of 2CLCs. We hypothesize that CTCF will be not only recruited to the DUX condensates but also exhibit a DUX-like binding pattern like CBP/p300. Dux overexpression increased CTCF binding at reorganized TAD (Fig. S5E), demonstrating CTCF’s role as a regulator during cohesin-mediated loop extrusion rather than merely serving as boundaries for intra-TAD chromatin interactions (Davidson et al., 2023). These CTCF containing loops exhibited co-enrichment of p300 (Fig. 5H), implicating a cooperative mechanism where phase-separated Dux condensates concentrate acetyltransferase activity to stabilize transcriptional hubs. The enrichment of CTCF induced by the phase separation of DUX facilitates the maintenance and generation of loops containing totipotency-like-associated SEs (Fig. 5I). In Dux^R70A^-expressing cells, the enrichment of CTCF (Fig. 5H) and the associated SE loops (Fig. 5J) are disrupted, highlighting the critical role of phase separation in enabling CTCF to enhance totipotency-like-related SE-promoter interactions and activate 2C genes (Fig. S5F and S5G).

Notably, Dux-driven chromatin reorganization diverges from pluripotency mechanisms. While OCT4 condensates dissolve TAD boundaries by displacing CTCF during cell reprogramming (Wang et al., 2021), Dux condensates recruit CTCF to reinforce intra-TAD interactions (Fig. 5K). This distinct behavior enables *de novo* loop formation between retrotransposon-derived SEs (e.g., MERVL/MT2) and 2C gene promoters, bypassing direct DUX binding.

### DUX condensates-driven super-enhancer assembly licenses retrotransposon activation and totipotency acquisition

**Figure 6.**
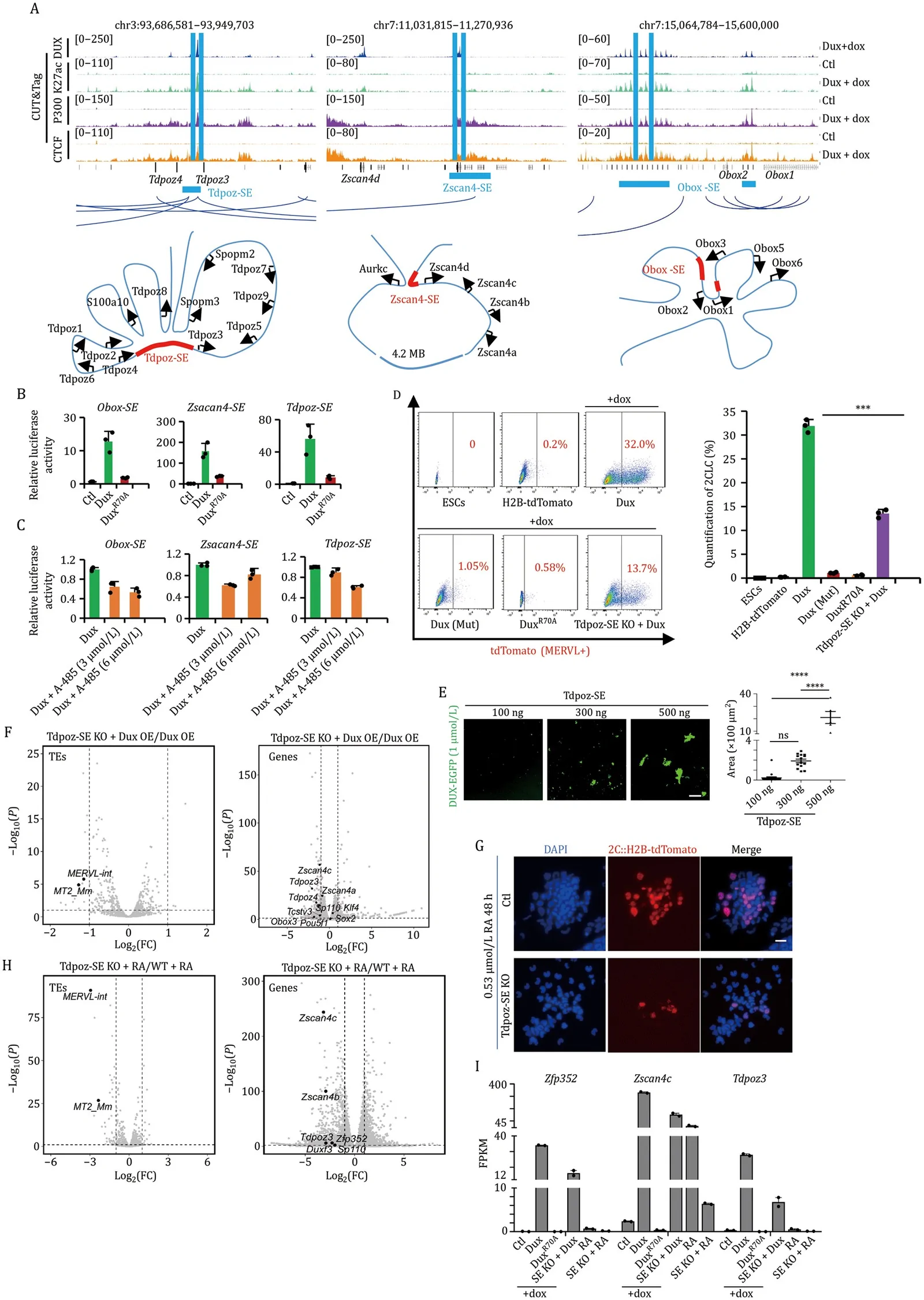
**DUX induced SEs are essential for pluripotent to totipotent transition**. (A) Illustration of chromatin loops detected at Tdpoz (left), Zscan4 (middle), and Obox (right) gene cluster. (B) Dual luciferase assays were conducted in the cells co-transfected with luciferase reporter driven by Obox-SE, Zscan4-SE, or Tdpoz-SE, along with overexpression constructs of DUX and DUX^R70A^. Data are presented as mean ± SD. (C) Dual luciferase assays were conducted in the cells co-transfected with luciferase reporter driven by Obox-SE, Zscan4-SE, or Tdpoz-SE, along with overexpression constructs of DUX after treatment with p300/CBP inhibitor A-458. Data are presented as mean ± SD. (D) Flow cytometry profiles of MERVL-positive cells after expressing Dux or phase separation deficiency DUX^R70A^ in the Tdpoz-SE knockout cells. (E) Phase separation analysis of EGFP-DUX in the presence of varying amounts of Tdpoz-SE DNA (100–500 ng) was conducted under conditions of 10% PEG8000 and 125 mmol/L NaCl (left panel). The right panel quantifies the area of EGFP-DUX puncta formed with Tdpoz-SE DNA. Data are presented as the mean ± SEM. ns, not significant; ****P < 0.0001 (*t*-test). Scale bars: 100 μm. (F) Volcano plots illustrate the TE (left panel) and DEGs (right panel) in Dux-overexpressed Tdpoz-SE knockout ESCs compared to Dux-overexpressed ESCs alone (*n* = 2 biological replicates). Noteworthy TEs and genes are highlighted. (G) Fluorescence microscopy images illustrate MERVL-positive cells in Tdpoz-SE knockout cells following a 48-hour treatment with 0.53 μM RA for 48 h (*n* = 3 biological replicates). Scale bars: 50 µm. (H) Volcano plots depict the transcriptional elements (TE, left panel) and differentially expressed genes (DEGs, right panel) in Tdpoz-SE knockout ESCs relative to control cells after 48 h treatment of 0.53 μmol/L RA (*n* = 2 biological replicates). Noteworthy TEs and genes are highlighted. (I) Plot illustrates the mean ± SD for fragments per kilobase per million (FPKM) values for *Zfp352*, *Zscan4c* and *Tdpoz3*.

The activation of endogenous retroviruses (MERVL) and 2C-specific genes is a hallmark of totipotency. To dissect how DUX condensates license this transcriptional program, we focused on SEs proximal to key 2C loci induced by DUX. A total of 107 SEs were identified, with the majority (53.2%, 90/169) linked to 2C genes (Fig. S6A), including Obox, Zscan4, and Tdpoz clusters (Fig. 6A). These SEs exhibited robust chromatin looping to their target promoters in Dux-overexpressing cells, accompanied by elevated CTCF binding, H3K27ac deposition, and p300 recruitment (Fig. 6A). Luciferase reporters driven by these SEs revealed that DUX, but not the phase separation-deficient Dux^R70A^, enhanced transcriptional activity by 20–160 fold (Fig. 6B). Inhibition of p300/CBP with A-485 abolished this activation (Fig. 6C), confirming that SE function depends on acetyltransferase recruitment via DUX phase separation.

Previous studies have reported that Obox and Zscan4 family genes activate totipotency (Ji et al., 2023; Zhang et al., 2019). Next, we focus on the Tdpoz-SE to directly assess the role of SEs in totipotency. Given that Tdpoz-SE is established through the phase separation of Dux, we investigated its impact on enhancing Dux condensation. Tdpoz-SE DNA enhanced DUX condensation *in vitro*, even at sub-saturating protein concentrations (Fig. 6E). This positive feedback loop—where SE DNA stabilizes DUX condensates, which in turn reinforces SE activity—ensures robust transcriptional activation of retrotransposons and 2C gene. Then, we deleted the Tdpoz-SE using CRISPR-Cas9 (Fig. S6B). Loss of Tdpoz-SE abrogated MERVL activation in Dux-expressing cells (Fig. 6D), mirroring the phenotype of Dux^R70A^ (Fig. S6C and S6D). RNA-seq analysis revealed that Tdpoz-SE knockout cells failed to upregulate some 2C genes, including *Zscan4c* and *Obox3*, while canonical pluripotency markers remained unaffected (Figs. 6F and S6E–G). Notably, Tdpoz-SE deletion impaired chemical reprogramming to 2CLCs by retinoic acid (RA) (Fig. 6G–I) or PlaB (Fig. S6H and S6J), underscoring the SE’s necessity for universal totipotency induction.

Collectively, these results establish that DUX-mediated phase separation licenses SE assembly at retrotransposon-rich loci, enabling spatial coordination of enhancer-promoter interactions and transcriptional bursting. This mechanism bypasses the need for direct DUX-DNA binding at distal targets, resolving the paradox of how DUX indirectly activates most of its transcriptional program during the pluripotency-to-totipotency transition.

### Conserved IDR-mediated phase separation underlies the functional conservation of DUX family proteins across species

**Figure 7.**
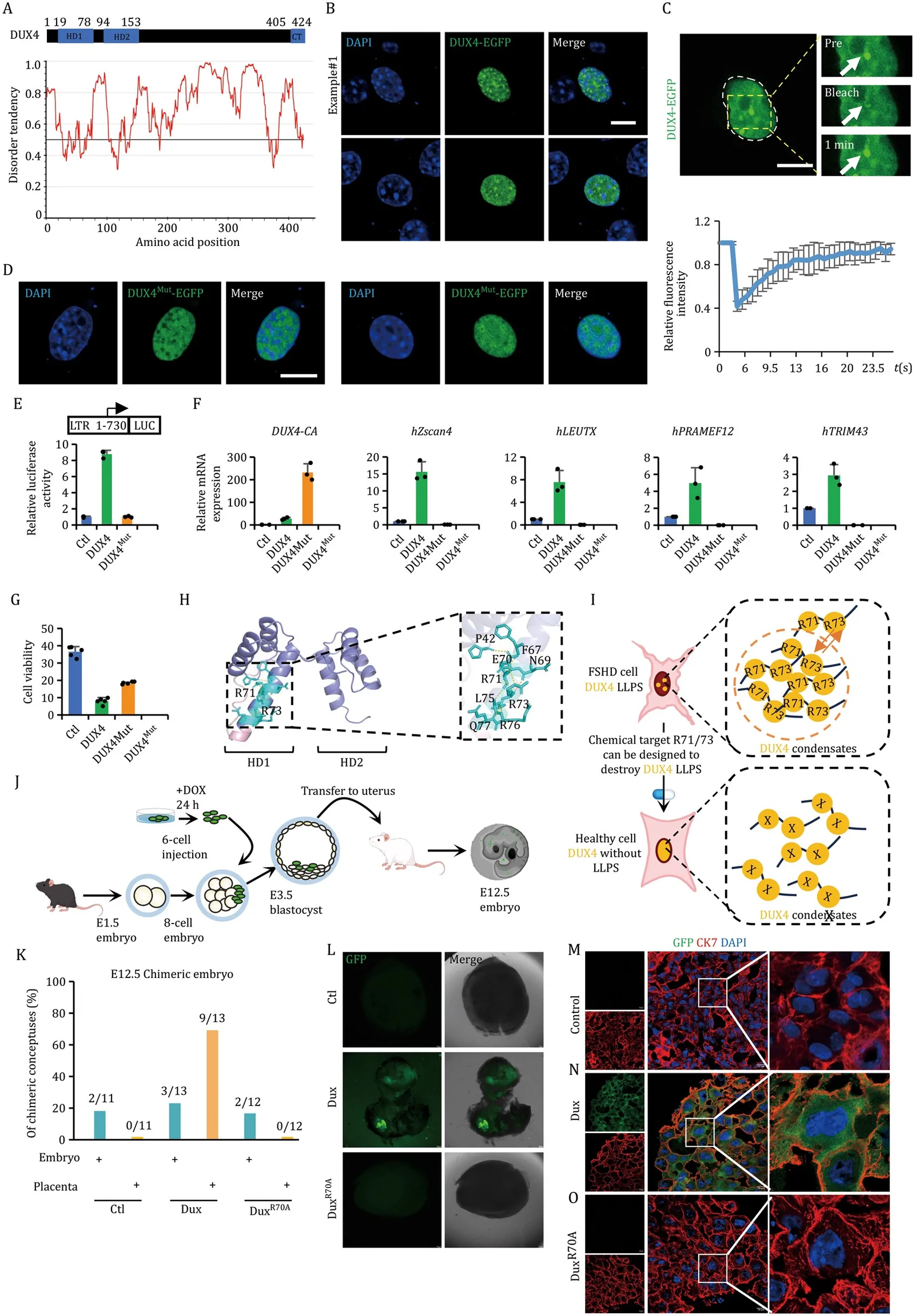
**Conservation of IDR and function in DUX proteins (human DUX4 and porcine DUXC)**. (A) Protein sequence analysis and disorder prediction (using IUPred) for the DUX4. The numbers above the sequence represent the positions of the amino acids (aa). A score exceeding 0.5 indicates regions predicted to be disordered. (B) Live-cell imaging of cells expressing EGFP-DUX4. Scale bar: 5 µm. (C) FRAP analysis of EGFP-DUX4 droplets in the cells was conducted by bleaching at positions indicated by white arrows. Quantification of fluorescence intensity during the FRAP assay is shown below (*n* = 8 cells). Scale bars: 5 μm. (D) Live-cell images indicated that EGFP-tagged DUX4 mutants were successfully expressed in transfected cells. Scale bars: 5 μm. (E) Dual luciferase assay was employed to detect MERVL activation induced by DUX4/DUX4 mutant vectors. (F) RT-qPCR analysis of DUX4 target genes, including *ZSCAN4*, *LEUTX*, *PRAMEF12*, and *TRIM43* upon overexpression of DUX4 and its mutant variants. DUX4-CA refers to a codon-optimized version of DUX4, designed to enhance translation efficiency in mammalian cells. (G) Human cells were transfected with plasmids expressing either wild-type DUX4 or its mutant variant, and cell viability was assessed 48 h post-transfection using the CCK-8 assay. (H) The views of Arg71 and Arg73 in HD1 are presented, highlighting their side chains that form base-specific contacts. Selected surrounding residues are displayed in stick representation. Hydrogen-bonding interactions are indicated by yellow dotted lines. (I) Strategy for drug development targets for FSHD induced by DUX4 phase separation. (J) The procedure involves injecting cells overexpressing Dux or Dux^R70A^ into eight-cell embryos, followed by *in vitro* culture until the blastocyst stage (E3.5). Subsequently, the embryos are transferred back to the uteri of recipient females and allowed to develop until E12.5. (K) The proportion of embryos and the number of chimeric embryos exhibiting contributions to the trophectoderm (TE), inner cell mass (ICM), and both TE and ICM at the E12.5 stage. (L) Micrographs illustrating the difference of the E12.5 placenta following the injection of cells overexpressing GFP-labeled Dux or the Dux^R70A^ mutant at eight-cell stage. Scale bars: 500 μm. (M–O) Immunofluorescence staining of the E12.5 placenta was performed after injecting cells overexpressing GFP-labeled wild-type Dux or the Dux^R70A^ mutant at the eight-cell stage. CK7 was used as a marker for the syncytiotrophoblast layer. Scale bars: 50 μm.

The evolutionary conservation of DUX-family transcription factors extends to their structural dependency on intrinsically disordered regions (IDRs) for phase separation and transcriptional activity. Sequence alignment of mouse Dux, human DUX4, porcine DUXC, and rat DUX revealed striking conservation of arginine residues (R70 and R72 in mouse Dux; R71/R73 in human DUX4) (Fig. S7A). Computational disorder prediction (IUPred2) confirmed that these arginine residues are highly conserved across species (Figs. 7A and S7B), suggesting a shared mechanism for LLPS.

Live-cell imaging demonstrated that EGFP-tagged human DUX4 and porcine DUXC formed dynamic nuclear condensates resembling those of mouse Dux (Figs. 7B and S7C). FRAP assays revealed rapid fluorescence recovery for both DUX4 and DUXC condensates (Figs. 7C and S7D), confirming their liquid-like properties. Given that DUX4 serves as a critical driver in the pathogenesis of human muscular dystrophy, we further investigated whether DUX4 are sufficient for phase separation by substituting the conserved arginine residues at positions R70 and R72 with alanine. Mutating conserved arginines (DUX4^R71A/R73A^) abolished condensate formation (Fig. 7D), mirroring the phase separation deficiency observed in mouse DUX^R70A^ (Fig. 2H), thus establishing weak interactions mediated by these residues as a universal driver of phase separation.

Functional assays across species underscored the necessity of IDR-mediated phase separation for transcriptional activation. Wild-type human DUX4 robustly activated a MERVL-driven luciferase reporter, whereas phase separation-deficient mutants (DUX4^R71A/R73A^) failed to induce transcription (Fig. 7E). Similarly, RT-qPCR confirmed that DUX4-dependent genes (*ZSCAN4*, *LEUTX*, *TRIM43*) were silenced in mutants (Fig. 7F). Strikingly, disrupting DUX4 condensates significantly reduced cytotoxicity in facioscapulohumeral muscular dystrophy (FSHD) models, rescuing cell viability (Fig. 7G). Structural modeling revealed that conserved arginines (R71/R73 in DUX4) form cation-π interactions and salt bridges with neighboring residues (Fig. 7H), stabilizing the conformation necessary for phase separation.

These findings highlight a unified mechanism wherein IDR-mediated phase separation is indispensable for the transcriptional and pathogenic functions of DUX-family proteins across mammals. The evolutionary preservation of this motif underscores its critical role in licensing totipotency and disease pathogenesis, offering a conserved target for therapeutic intervention in conditions like FSHD (Fig. 7I).

### Deficiency in DUX condensates abrogates embryonic developmental potential

To assess the physiological relevance of DUX phase separation *in vivo*, we investigated the developmental capacity of phase separation-deficient DUX in mouse embryos. GFP-labeled embryonic stem cells (ESCs) overexpressing wild-type Dux or the phase separation-defective mutant Dux^R70A^ were microinjected into eight-cell stage embryos, followed by tracing their contributions to embryonic and extraembryonic lineages (Fig. 7J). At the blastocyst stage (E3.5), Dux-expressing cells robustly contributed to both the inner cell mass (ICM; SOX2 positive) and trophectoderm (TE; CDX2 positive), with 42.2% (19/45) of embryos exhibiting dual-lineage chimerism (Fig. S7E–H). In stark contrast, Dux^R70A^ cells failed to integrate into the TE, localizing exclusively to the ICM (0/35 embryos; Fig. S7H).

To evaluate post-implantation potential, chimeric embryos were transferred to pseudo-pregnant females and analyzed at E12.5. Remarkably, 69.2% (9/13) of embryos injected with Dux-expressing ESCs exhibited GFP^+^ cells in placental tissues (Fig.7K and 7L), confirmed by co-staining with the syncytiotrophoblast marker CK7 (Fig. 7M–O). Conversely, Dux^R70A^ cells showed no contribution to placental development (0/12 embryos; Fig. 7K), mirroring the restricted lineage potential of pluripotent cells. These findings demonstrate that DUX condensates are essential for conferring expanded developmental plasticity, enabling contribution to both embryonic and extraembryonic lineages—a hallmark of totipotency.

## Discussion

The pluripotent mouse ESCs can cycle into a totipotent state, wherein a significant proportion of the transcriptome characteristic of two-cell-stage embryos becomes activated (Macfarlan et al., 2012). In this work, we analyze the published data and identify mouse Dux and its human homolog DUX4 as playing crucial roles in totipotency regulation both *in vivo* and *in vitro* (De Iaco et al., 2017; Hendrickson et al., 2017; Whiddon et al., 2017, Yang et al., 2020). Although Dux has been reported to activate MERVL/2C gene expression, only three investigations have specifically focused on the function of Dux itself. These studies primarily utilized Dux ChIP-seq data to show that DUX binds to MERVL/LTR elements, thereby activating a substantial proportion of 2C-specific genes (De Iaco et al., 2017, Hendrickson et al., 2017, Whiddon et al., 2017). However, how Dux activates approximately 75% of genes without direct binding remains to be elucidated (Iturbide and Torres-Padilla, 2017). While one possible explanation involves indirect regulatory mechanisms such as tethering, we emphasize that this does not represent the primary mode of DUX-mediated regulation. DUX is fully capable of directly binding canonical motifs in promoters or enhancers, as well as retrotransposon elements, and it also activates downstream transcription factors and chromatin regulators that modulate additional targets through conventional indirect pathways. Regardless of the exact mechanism, an essential feature is that Dux rapidly activates a broad spectrum of 2C-specific genes within a short time frame. The strong correlation observed between Dux-induced 2CLCs and two-cell-stage embryos by ATAC-seq analysis (Hendrickson et al., 2017) suggests that this process may involve global chromatin reorganization regulated by Dux, and we emphasize that DUX achieves this precise, genome-wide regulation through its phase-separation capability. Recruitment of CBP/p300 by Dux can induce a global reorganization of H3K27 acetylation thereby rendering previously inaccessible chromatin regions permissive for transcription. Although previous studies have shown that CBP/p300 physically interacts with DUX4 via CTD (Choi et al., 2016), our study proposes an additional mechanism: the ability of Dux to form condensates through phase separation may facilitate the functional recruitment or spatial enrichment of CBP/p300 at specific genomic loci. Furthermore, the phase separation of Dux facilitates spatially defined acetylation of MERVL and 2C genes, a process that cannot be achieved through CTD interaction alone.

FSHD is caused by the abnormal derepression of the myotoxic transcription factor DUX4 (Lemmers et al., 2010). Since the identification of DUX4 as the causal gene in FSHD, there has been significant interest in developing therapeutic strategies targeting DUX4 inhibition. However, the post-transcriptional regulation and functional mechanisms of the DUX4 protein remain largely elusive. To date, no approved treatments directly interfere with the ability of the DUX4 protein to activate downstream genes, and current management is primarily supportive. This study represents the first investigation into the importance of the organizational form of DUX4 protein for its function. Notably, we found that a conserved arginine residue within the DUX4 IDR determines both the phase separation properties and the transcriptional activation of myotoxic genes. Further research is needed to identify the interaction partners and DNA binding that are regulated by the DUX4 condensates. Additionally, investigating chemicals that disrupt DUX4 condensates may offer potential therapeutic avenues for FSHD (Fig. 7I).

LLPS has been implicated in many biological processes (Alberti et al., 2019; Ding et al., 2024). During the maintenance of pluripotency in embryonic stem cells, multiple master pluripotent transcription factors like Oct4, Nanog, and Sox2 have been reported to undergo biomolecular condensation via their activation domain (AD) to activate gene expression (Boija et al., 2018; Wang et al., 2021). While some of the transcription factors exhibit low phase separation property, they can be incorporated into phase-separated droplets formed by coactivators like Med1/BRD4 at SEs, leading to transcriptional bursting of SE-driven genes. However, there are differing perspectives on AD-dependent phase separation in transcriptional regulation. Multivalent interactions mediated by the AD only enhance the transcriptional activation capacity of a TF by increasing its residence time in the chromatin-bound state and facilitating the recruitment of coactivators independently of phase separation. To find out other domains, such as Homeobox, in the transcriptional hub establishment will help us understand the phase separation function in the transcriptional regulation. Furthermore, our data provide clear mechanistic insight into the “chicken-and-egg” question regarding the interplay between condensate formation, chromatin remodeling, and DNA binding. While it remains challenging to definitively resolve the chronological sequence of these events on a millisecond timescale, our findings suggest a hierarchical model where DUX-mediated phase separation serves as the primary driver. We propose that DUX condensates create a “hyper-accessible” chromatin environment by locally enriching coactivators like CBP/p300, which in turn acts as a prerequisite for the subsequent recruitment of downstream factors.

Notably, DUX-driven condensates operate distinctly from pluripotency mechanisms within 3D genome architecture. While OCT4 condensates dissolve TAD boundaries by displacing CTCF during cell reprogramming (Wang et al., 2021), DUX recruits CTCF to stabilize enhancer-promoter loops—a divergence that may reflect the transient, explosive transcriptional activation required for zygotic genome activation. Our data suggest that this recruitment is not merely passive occupancy of opened chromatin; rather, the DUX condensate likely provides a platform that facilitates the stabilization or functional loading of CTCF at these newly accessible sites. This dichotomy underscores how phase separation is tailored to meet the unique demands of distinct developmental states.

In conclusion, this work redefines totipotency as a phase-separated transcriptional state, orchestrated by DUX through dynamic biomolecular condensation. By bridging chromatin architecture, retrotransposon activation, and lineage plasticity, DUX condensates unlock the full developmental potential of the early embryo–a paradigm with far-reaching implications for regenerative medicine and disease therapy.

## Supplementary Material

pwag014_Supplementary_Data

## Data Availability

The accession number for the data reported in this paper is NCBI Gene Expression Omnibus (GEO): GSE177058, GSE168688, GSE224300, GSE66581, GSE159623, GSE44288, GSE125238, GSE222636. Data sets generated in this study have been deposited in GEO with accession numbers GSE325140 (RNA-seq), GSE325141 (ATAC-seq), GSE325142 (CUT&Tag), and GSE325205 (Hi-C).

## Artificial intelligence (AI)

During the preparation of this work, the author(s) used ChatGPT to perform grammar checks and improve the overall language and readability of the manuscript.

## Abbreviations

1,6-Hex: 1,6-hexanediol
2C: two-cell embryo
2CLC: two-cell-like cell
3D: three-dimensional
AD: activation domain
APA: aggregate peak analysis
ATAC-seq: assay for transposase-accessible chromatin using sequencing
BRD4: bromodomain-containing protein 4
BSA: bovine serum albumin
CBP: CREB-binding protein
CCK-8: Cell Counting Kit-8
cDNA: complementary DNA
ChIP-seq: chromatin immunoprecipitation sequencing
Co-IP: co-immunoprecipitation
CRISPR: clustered regularly interspaced short palindromic repeats
CTCF: CCCTC-binding factor
CTD: C-terminal domain
CUT&Tag: cleavage under targets and tagmentation
DAPI: 4',6-diamidino-2-phenylindole
DEG: differentially expressed gene
DMEM: Dulbecco’s modified eagle medium
dox: doxycycline
DPPA2/4: developmental pluripotency-associated protein 2/4
DUX: double homeobox transcription factor
DUX4: double homeobox 4 (human)
DUXC: double homeobox C (porcine)
E2C: early two-cell embryo
E3.5: embryonic day 3.5
E12.5: embryonic day 12.5
EDTA: ethylenediaminetetraacetic acid
EGFP: enhanced green fluorescent protein
EMSA: electrophoretic mobility shift assay
ESC: embryonic stem cell
FBS: fetal bovine serum
FE: fold-enrichment
FPKM: fragments per kilobase of transcript per million mapped reads
FRAP: fluorescence recovery after photobleaching
FSHD: facioscapulohumeral muscular dystrophy
GATA3: GATA binding protein 3
GFP: green fluorescent protein
GSEA: gene set enrichment analysis
H3K27ac: acetylation of lysine 27 on histone H3
H3K4me1: monomethylation of lysine 4 on histone H3
HEK293T: human embryonic kidney 293 T cells
HERVL: human endogenous retrovirus L
Hi-C: high-throughput chromatin conformation capture
HOMER: hypergeometric optimization of motif enrichment
HRP: horseradish peroxidase
ICM: inner cell mass
IDR: intrinsically disordered region
IgG: immunoglobulin G
IPTG: isopropyl β-D-1-thiogalactopyranoside
LIF: leukemia inhibitory factor
LLPS: liquid–liquid phase separation
LTR: long terminal repeat
MAPQ: mapping quality
Med1: mediator complex subunit 1
MERVL: murine endogenous retrovirus L
mESC: mouse embryonic stem cell
mRNA: messenger RNA
MT2: mammalian apparent LTR retrotransposon 2
NEAA: non-essential amino acids
OCT4: octamer-binding transcription factor 4
OE: overexpression
P-T: pluripotency-to-totipotency
p300: E1A binding protein p300
PAGE: polyacrylamide gel electrophoresis
PBS: phosphate-buffered saline
PBST: phosphate-buffered saline with Tween 20
PCA: principal component analysis
PCR: polymerase chain reaction
PFA: paraformaldehyde
PLAAC: prion-like amino acid composition
PrLD: prion-like domain
PVDF: polyvinylidene fluoride
RA: retinoic acid
RIPA: radioimmunoprecipitation assay
RNA-seq: RNA sequencing
ROSE: ranking of super-enhancers
RPM: reads per million
RT-qPCR: reverse transcription quantitative polymerase chain reaction
SD: standard deviation
SDS: sodium dodecyl sulfate
SE: super-enhancer
SEA: simple enrichment analysis
SEM: standard error of the mean
shRNA: short hairpin RNA
SOX2: SRY-box transcription factor 2
TAD: topologically associating domain
TBST: Tris-buffered saline with Tween 20
TE: typical enhancer
TF: transcription factor
UVR: ultraviolet radiation
WGCNA: weighted gene co-expression network analysis
ZFP352: zinc finger protein 352
ZGA: zygotic genome activation
ZSCAN4: zinc finger and SCAN domain containing 4
