# Supplementary material for: Phase separation of DUX family proteins drives totipotent-like state via 3D genome reorganization and retrotransposon activation
Source: Protein Cell. 2026 Mar 15;17(9):858–75. doi: 10.1093/procel/pwag014 (PMC13521275; doi:10.1093/procel/pwag014)
Supplement: Supplementary file 1 [file pwag014_supplementary_data.zip › PAC-25784-YF-Supplementary materials.pdf]

Materials and methods

Cell lines and culture conditions

Feeder-free mouse embryonic stem cells (mESCs) were cultured on 0.1% gelatin (Biosharp, BS160) coated plates and in ESM medium: DMEM (Corning, 10-013-CV) supplemented with 15% fetal bovine serum (FBS, Biochannel, BC-SE-FBS01), 1000 units/mL recombinant leukemia inhibitory factor (LIF, Sino Biological, 50756-MNAH), 0.1 mM 2-mercaptoethanol (Thermo Fisher Scientific, 21985023), 2 mM L-glutamine, 0.1 mM MEM non-essential amino acids (NEAA, Gibco, 11140076). HEK293T/NIH3T3 cells were grown in DMEM supplemented with 10% FBS (Biochannel, BC-SE-FBS07). All cells were cultured in normal conditions (37°C and 5% CO_2).

Lentiviral transduction and generation of stable cell lines

Lentiviruses were generated by transfecting HEK293T cells with the pCW57-MCS1-P2A-MCS2-mDux plasmid (Addgene, 138320) along with the packaging plasmids pMD2.g and psPAX2. The culture medium was replaced with 2 mL of fresh medium containing 15% fetal bovine serum (FBS). Supernatants containing virus particles were harvested at 24-, 48-, and 72-hours post-transfection and filtered through a 0.45 µm filter (Biosharp, BS-PES25-45-S). For infection, the viral supernatant supplemented with 8 µg/mL polybrene (Beyotime Biotechnology, C0351) was added to cell cultures at approximately 50% confluence for 12 hours. Following this period, the medium was replaced with fresh complete medium. At 48 hours post-infection, G418 was introduced into the medium to selectively eliminate uninfected cells over a one-week period. Using this protocol, ESC lines expressing Dux and the Dux^{R70A} mutant were successfully established.

Bacteria cells growth

Cloning of plasmid vectors was conducted in DH5α chemically competent cells (Tsingke Biotechnology, TSC-C14). Transfected cells were cultured on agar plates containing 100 mg/ml ampicillin for 24 hours at 37°C, after which several colonies were selected for sequencing and further expansion. Transfected E. coli cultures were grown overnight in LB-carb media at 37°C with shaking.

Doxycycline treatment of cells

Following a 24 hours incubation period post-plating, the cells were exposed to doxycycline (4 µg/mL; MCE, HY-N0565B) for an additional 24 hours to induce gene expression.

CRISPR knockout of Tdpoz-SE in ESCs

The knockout (KO) embryonic stem cell (ESC) line was generated using the CRISPR/Cas9 gene-editing technology. Specifically, ESCs were transfected with a puromycin-resistant pSpCas9(BB)-2A-Puro (PX459) V2.0 vector containing a guide RNA targeting the core sequence of Tdpoz-SE. Following transfection and drug selection, single-cell clones were isolated and expanded. A total of twenty-four clones were selected for genomic DNA extraction, and KO clones were subsequently validated through Sanger sequencing. CRISPR gRNAs that were inserted into PX459 were provided in Table S2.

Cell immunofluorescence staining and microscopy

Cells cultured on glass coverslips (Citotest Labware Manufacturing Co., Ltd.) were rinsed once with PBS, fixed with 4% paraformaldehyde (PFA) for 15 minutes, and permeabilized with 0.5% Triton X-100 (Sigma, 9036-19-5) for 10 minutes. Following three 5-minute washes with PBS, the cells were blocked with 10% fetal bovine serum (FBS; Biochannel, BC-SE-FBS07) at 37°C for 30 minutes. The cells were then incubated overnight at 4°C with the specified primary antibodies. After three additional 5-minute PBS washes, the cells were incubated with secondary antibodies for 1 hour in the dark at room temperature. Subsequently, the cells underwent three more PBS washes, and nuclei were counterstained with 1 μM 4',6-diamidino-2-phenylindole (DAPI; Beyotime Biotechnology). Finally, the cells were rinsed three times with PBS and mounted using a mounting medium. Images were captured using a laser scanning confocal microscope (FV3000, Olympus) equipped with a ×60 oil immersion objective. The antibodies used for immunofluorescence staining included: anti-H3K27ac (Activemotif, 39034), anti-H3K4me1 (Cell Signaling Technology, 5326), and anti-GFP (Santa Cruz Biotechnology, sc-9996).

Colocalization analysis

For the colocalization images from the same cells, we extracted the intensity values of each pixel within the selected regions for each channel using an ImageJ macro. Subsequently, we utilized GraphPad Prism to generate line graphs representing the fluorescence intensity of each pixel. In each experiment, we analyzed three biologically independent replicates.

Flow cytometry

ESCs expressing the tdTomato reporter were analyzed and sorted using a BD FACSAria III instrument (excitation wavelength: 561 nm). ESCs that did not express the tdTomato reporter served as negative controls. Data analysis was performed using FlowJo software (version 10.8.1).

Protein expression and purification

cDNA encoding the proteins of interest were cloned into the pET28a expression vector, which contains a His-tag and EGFP. Sanger sequencing was conducted to confirm the accuracy of the inserted sequences. For large-scale protein expression, the plasmids were transformed into chemically competent E. coli BL21 (DE3) cells (Tsingke Biotechnology, TSC-E01). The bacterial cultures were prepared as follows: A single colony was inoculated into LB medium supplemented with kanamycin (Solarbio, K8020) and incubated overnight at 37°C. Subsequently, the culture was diluted 1:30 in 300 mL of fresh LB medium containing kanamycin and grown at 37°C for approximately 5 hours until the OD600 reached 0.6-0.8. At this optical density, the bacteria are in the logarithmic growth phase, ensuring optimal activity and resistance to IPTG toxicity during protein expression. Induction was performed by adding IPTG (Solarbio, I1020-5) to a final concentration of 0.3 mM, followed by continued incubation at 16°C for 18 hours. Protein purification was carried out according to the manufacturer's instructions using the Protein Purification Kit (Cwbio, CW0894S). The recombinant GFP fusion proteins were concentrated using Amicon Ultra 30 KDa centrifugal filters (Millipore, UFC803024). Protein concentrations were quantified using a NanoDrop 2000 spectrophotometer (Thermo Scientific), and samples were stored at -80°C.

In vitro droplet assay

Different amounts of the recombinant protein were added to the droplet formation buffer, which comprised 50 mM Tris-HCl pH 7.5 (Thermo Fisher, 15567-027), 10% glycerol (Sigma, G5516), 1 mM DTT (Sigma, D9163), 20% PEG8000 (Sigma, H-209Z-0T968), and 100 mM NaCl (Sigma, S5150). All preparation steps were conducted at 4°C to preserve protein activity. The protein solution was promptly loaded onto slides for microscopic imaging using a Nikon microscope. For SE-facilitated DUX aggregation, DNA motifs of varying qualities were individually added to the droplet formation buffer. The Tdpoz-SE sequence was determined using ROSE software and integrated with the DUX motif sequence analyzed by Homer software, resulting in the Tdpoz-SE probe sequence as follows:

gagagctgggagtatctgaactaattgaatcactgtagttatttctgtgtcaatttgaggccaagcagagcagttcaggaaaaactgacagaactcagattgaatcagttaccttgagaggagccttaaatcaaccaaccagcgctcagaaggtgaatcctgccaattaaagtgattgaattgagaagcatttagaatatcc

Puncta analysis

To quantify the puncta in cells, confocal microscopy was employed with consistent parameters across all experimental groups (both with and without 1,6-hexanediol treatment). The number and area of puncta were subsequently analyzed using ImageJ software.

Luciferase assay

The core sequences of Tdpoz-SE, Zscan4-SE, and Obox-SE were synthesized by Tsingke Biological Technology Co., Ltd. (Wuhan, China) and subsequently inserted into the pGL3-promoter vector (Promega) to generate pGL3-promoter-Tdpoz4-SE, pGL3-promoter-Zscan4-SE, and pGL3-promoter-Obox-SE constructs. These constructs were transfected in triplicate using Lipo8000 alongside 50 ng of pRL-TK and 200 ng of either the Dux or Dux (Mut) ectopic expression vectors. Luciferase and Renilla activities were measured 48 hours post-transfection using the Dual-Glo Luciferase Assay Kit (TransGen Biotech, FR201-01-V2), according to the manufacturer's instructions. All luciferase activities were normalized relative to the Renilla activity within the same sample.

Cell viability assay

Cell Counting Kit-8 (CCK-8) assays (Beyotime Biotechnology, C0037) were conducted to evaluate cell viability. Human embryonic kidney 293T (HEK293T) cells were seeded in 96-well plates and subsequently transfected with either DUX4 or its mutant form, $DUX4^{Mut}$. To induce gene expression, doxycycline (dox) was administered 24 hours post-transfection. Following this, 10 μL of CCK-8 reagent was added to each well and the plate was incubated at 37 °C for one hour. The absorbance at 450 nm was then measured using a microplate reader (Thermo Fisher Scientific, model Varioskan LUX).

Quantitative real-time PCR (RT-qPCR)

To quantify mRNA expression levels, total RNA was extracted from samples using Trizol reagent. Subsequently, 1 μg of total RNA was reverse-transcribed into cDNA using a reverse transcriptase kit (iScience, EG15133S). Gene expression levels were normalized to those of beta-Actin. The primers for qPCR are provided in Supplementary information, Table S2.

Western blot analysis

The cells were lysed in RIPA lysis buffer (Beyotime Biotechnology, P0013B) supplemented with protease and phosphatase inhibitors. Proteins from the lysates were separated by SDS-PAGE and

subsequently transferred onto a PVDF membrane (Millipore, ISEQ00010). Immunoblotting was performed using primary antibodies of interest incubated overnight at 4°C. The membranes were washed three times for 10 minutes each with TBST, followed by incubation with HRP-conjugated secondary antibodies (Boster Biological Technology Co., Ltd.) for 1 hour at room temperature. After washing the membranes three times with TBST, they were incubated in detection solution (Mishushengwu, MI00607A) and visualized using a chemiluminescence imaging system (Tanon, #5800).

Fluorescence recovery after photobleaching (FRAP) assay

For FRAP experiments conducted in cells, DUX-EGFP was transfected into NIH3T3 or ESCs and subsequently induced with doxycycline for 24 hours. For FRAP analysis of purified DUX-EGFP protein, the protein was first expressed in a prokaryotic system, purified, and then immobilized on glass slides. Fluorescence images of EGFP were captured using super-resolution microscopy equipped with a 100x oil-immersion objective lens (A1R N-SIM N-STORM). The fluorescence intensity of bleached regions at each time point was normalized to both the background fluorescence intensity and that of adjacent unbleached regions. Fluorescence intensities within the condensates were quantified using NIS-Elements software.

1,6-hexanediol treatment

As previously described (Liu et al., 2021), to evaluate the sensitivity of protein aggregates to 1,6-hexanediol treatment, media containing various concentrations of 1,6-hexanediol (Macklin, H810886) were prepared and pre-warmed in a 37°C incubator. For treatment, the existing medium in each dish was discarded, and the pre-warmed medium with different concentrations of 1,6-hexanediol was added. The dishes were then immediately placed back into the incubator. Cells treated with 1,6-hexanediol were promptly utilized for subsequent experiments. For imaging experiments, cells were treated with 3% 1,6-hexanediol for 7 minutes prior to microscopy. For functional assays, cells were exposed to 0.75% 1,6-hexanediol continuously throughout the 24 hours DUX induction period.

Electrophoretic Mobility Shift Assay (EMSA)

Biotin-labeled single-stranded oligonucleotides containing the DUX binding motif on MERVL were mixed and annealed into a double-stranded probe using a temperature gradient from 95°C to 25°C. Binding reaction mixtures were assembled by sequentially adding: nuclease-free water, binding

buffer (Thermo Fisher Scientific, 20148A), the probe (with or without unlabeled competitor probe as indicated), and an equal amount of purified DUX or DUX^{R70A} protein. Reactions were mixed gently and incubated at 37°C for 30 minutes in a PCR machine. Immediately after incubation, samples were loaded onto a pre-run native polyacrylamide gel in an ice bath and electrophoresed at 80V for 90 minutes in cold 0.5x TBE buffer. The resolved complexes were then transferred onto a nylon membrane in cold 0.5x TBE. Following transfer, the membrane was immediately cross-linked under UV light for 25 minutes. Subsequent detection was performed using a chemiluminescent EMSA kit (Beyotime Biotechnology, GS009). The membrane was blocked, incubated with an appropriate amount of HRP-conjugated streptavidin, and washed according to the manufacturer's protocol. Finally, signals were visualized using a chemiluminescence imaging system (Tanon, #5800). The sequences of oligonucleotides used for EMSA are listed in Supplementary Table S2.

Microinjection and chimeric assay

To generate chimeric blastocysts via microinjection, cells from the Dux-activated ESC line, Dux^{R70A}-activated ESCs, and a control group were separately injected into 8-cell stage recipient embryos. Prior to injection, these cells were labeled with GFP. The injected embryos were cultured in KSOM medium under standard conditions (37°C, 5% CO_2) for 24 hours to develop into chimeric blastocysts. GFP-positive cells were identified using immunofluorescence stereomicroscopy, followed by immunofluorescence staining. Subsequently, the chimeric blastocysts were transferred to the uterine horns of pseudo-pregnant ICR females at 2.5 days post coitum. At embryonic day 12.5 (E12.5), the fetuses and placentas were dissected, and GFP-positive cells were localized using immunofluorescence stereomicroscopy. The placentas were isolated from E12.5 fetuses, embedded, frozen, sectioned (10 µm thickness), and subjected to immunofluorescence staining on the frozen sections.

E3.5 blastocyst immunofluorescence staining

The chimeric blastocysts were transferred from KSOM culture to PVA/PBS using oral pipettes three times, then fixed in 4% PFA at room temperature for 15 minutes. They were washed three times with 1% BSA/PBS, followed by permeabilization in 0.3% Triton X-100 for 15 minutes. After additional washes with 1% BSA/PBS, the samples were blocked at room temperature with 3% BSA/PBS for 1 hour. Primary antibodies (SOX2/GATA3) were incubated overnight at 4°C. Following three washes with 1% BSA/PBS, secondary antibodies were incubated at room

temperature for 45 minutes. After further washing with 1% BSA/PBS, the samples were stained with DAPI for 5 minutes and finally transferred to 1% BSA/PBS for observation under a fluorescence microscope.

Immunofluorescence staining

The sections were dried overnight at room temperature. The OCT-embedded samples were first immersed in PBS for 1 hour, and then permeabilization with 0.3% Triton X-100 for 20 minutes. The film was placed in citric acid buffer at 95°C for antigen retrieval for 10min. After cooling to room temperature for 1 hour, the sections were washed twice with PBST. Blocking was carried out using 3% BSA at room temperature for 1 hour. Primary antibody (CK7) was incubated overnight at 4°C. After cleaning with PBST for 2 times, incubate with secondary antibody at room temperature for 1 hour. After washing twice with PBST, the sections were stained with DAPI for 5 minutes and subsequently transferred to PBS for observation under a fluorescence microscope.

CUT&Tag

CUT&Tag experiments were all performed with two biological replicates per condition. One million ESCs were cultured for 24 hours and subsequently induced with doxycycline (4 μg/mL) for an additional 24 hours. Following induction, the cells were washed once with PBS and sorted by flow cytometry to collect 100,000 MERVL-positive and MERVL-negative cells for CUT&Tag analysis using the Novoprotein N259-YH01 kit. ConA-streptavidin beads were conjugated with live cells and incubated for 2 hours at room temperature in binding buffer with antibodies of interest diluted 1:100. Goat anti-rabbit IgG H&L (Novoprotein, N269) was then incubated for 1 hour at room temperature at a dilution of 1:200. Subsequently, pA-Tn5 transposase was incubated for 1 hour at room temperature at a concentration of 1:100. After washing steps and elution from the beads, PCR amplification was performed for 17 cycles. The samples were purified and prepared for sequencing on the Illumina NovaSeq 6000 platform using 150-bp paired-end reads. Raw sequencing reads underwent quality control and adapter trimming using Trim Galore software (version unspecified). High-quality reads were aligned to the UCSC mm10 reference genome using Bowtie2 (version 2.4.5) with stringent parameters (--end-to-end, --very-sensitive, --no-mixed). Picard (version 2.25) was employed to remove duplicate reads from BAM files, and Samtools (version 1.9) was used to filter reads with MAPQ values greater than 30 for high-quality alignment results. The filtered BAM files were indexed using Samtools. Deeptools (version 3.5.5) was utilized to generate bigWig files and

calculate protein enrichment levels. The antibodies used for CUT&Tag included: anti-H3K27ac (Activemotif, 39034), anti-FLAG (Cell Signaling Technology, 14793), anti-p300 (Cell Signaling, Technology 54062), and anti-CTCF (Cell Signaling Technology, 3418).

ChIP-seq data analysis

Following the removal of low-quality reads from the ChIP-seq raw data using Trim Galore, the processed datasets were aligned to the mouse genome (build mm10) with Bowtie2 (version 2.4.5). Picard (version 2.25) was employed to eliminate duplicate reads in the BAM files, and Samtools was utilized to index the alignment results. MACS2 was subsequently applied to identify peaks for each ChIP sample, followed by the generation of FE-normalized bigWig files to compare the average density of transcription factors (TFs) across different genomic regions. Finally, DeepTools (version 3.5.5) was used to assess the enrichment levels of TFs.

Super enhancer identification

Totipotency- and pluripotency-related super-enhancers (SEs) were identified using the ROSE algorithm (version 1.3.1) with parameters -s 12500 and -t 2500, as described in the published study (Hnisz et al., 2013). Additionally, genes associated with these super-enhancers were retrieved from the ROSE output.

Enrichment analysis of transcription factors on SE

We employed the MACS2 version 2.2.7.1 (model-based analysis of ChIP-seq) peak calling algorithm to identify regions enriched for transcription factor ChIP-seq signals relative to background. A q-value threshold of 0.01 was applied consistently across all data sets to determine significant enrichment. Subsequently, bigwig files were generated using MACS2's bdgcmp subfunction and normalized in fold-enrichment (FE). The ComputeMatrix tool was utilized to quantify the enrichment levels of each transcription factor at super-enhancers (SE), with the results visualized as boxplots. Statistical significance was assessed using the Wilcoxon rank-sum test.

Enrichment analysis of ATAC-seq/CUT&Tag

The bigwig files for ATAC-seq and CUT&Tag were generated using bamCoverage, with normalization performed according to the RPKM method. The enrichment levels of each transcription factor on super-enhancers (SE) or typical enhancers (TE) were quantified using computeMatrix, followed by visualization of the results. Statistical significance was assessed using the Wilcoxon rank-sum test (wilcox.test).

Enrichment analysis of TF binding motif

The majority of transcription factor motif matrices were obtained from the JASPAR database. For those transcription factors for which direct motif matrix data were unavailable, we processed their ChIP-seq datasets using HOMER to derive the corresponding motif matrices. The totipotency super-enhancers identified in MERVL-positive cells were utilized as input for the SEA algorithm on the MEME suite. Consequently, we obtained the enrichment rankings and P-values associated with these super-enhancers.

RNA-seq and data analysis

RNA-seq was performed with two independent biological replicates. For ESCs, total RNA was isolated using the Trizol Reagent (Invitrogen Life Technologies), after which the concentration, quality and integrity were determined using a NanoDrop spectrophotometer (Thermo Scientific). Three micrograms of RNA were used as input material for library preparation. Products were purified (AMPure XP system) and quantified using the Agilent high sensitivity DNA assay on a Bioanalyzer 2100 system (Agilent). The sequencing library was then sequenced on NovaSeq 6000 platform (Illumina) Shanghai Personal Biotechnology Cp. Ltd. Trim Galore was employed to filter out low-quality reads from the raw dataset. The filtered reads were subsequently aligned to the mm10 mouse genome using the HISAT2 aligner. FeatureCounts was then utilized to generate an expression matrix and to calculate Fragments Per Kilobase of transcript per Million mapped reads (FPKM) values for each gene, thereby quantifying gene expression levels.

ATAC-seq and data analysis

ATAC-seq experiments were all performed with two biological replicates per condition. The ATAC-seq libraries were constructed according to previous studies (Corces et al., 2017). ESCs were transfected with Dux, Dux (Mut), and empty plasmids, respectively, and induced with doxycycline (4 μg/mL) for 24 hours. Samples was collected, and the cell suspension was prepared. Cell membrane was lysed to obtain the nucleus. Then, Tn5 transposase was added to cut open DNA. The DNA fragments cut by the enzyme were amplified by PCR and then sequenced on the Illumina NovaSeq 6000 platform in a 150-bp paired-end format. Raw sequencing reads underwent quality control and adapter trimming using Trimmomatic (Version 0.40). High-quality reads were aligned to the UCSC mm10 reference genome using Bowtie2 (version 2.4.5) with stringent alignment parameters (--end-to-end, --very-sensitive, --no-mixed). Picard (version 2.25) was employed to

remove duplicate reads from BAM files, followed by extraction of reads with MAPQ values exceeding 30 using Samtools (version 1.9) to ensure high-quality alignments. The results were indexed using Samtools, and Deeptools (version 3.5.5) was utilized to generate bigWig files and calculate ATAC signal enrichment.

Hi-C

In situ Hi-C experiments were conducted following the protocol described by previous studies (Rao et al., 2014). The experimental procedures and library sequencing were performed by Frasergen Bioinformatics Co., Ltd. (Wuhan, China). Cells were cross-linked with 1% formaldehyde for 10 minutes at room temperature and quenched with glycine to a final concentration of 0.125 M for 5 minutes. Subsequently, the cross-linked cells were lysed. Endogenous nuclease activity was inactivated using 0.3% SDS, followed by digestion of chromatin DNA with 100 units of MboI (NEB). Biotin-14-dCTP (Invitrogen) was used to label the digested DNA, which was then ligated with 50 units of T4 DNA ligase (NEB). Cross-links were reversed, and the ligated DNA was extracted using the QIAamp DNA Mini Kit (Qiagen) according to the manufacturer's instructions. Purified DNA was sheared into fragments ranging from 300 to 500 base pairs, blunt-end repaired, A-tailed, and adaptors added. Further purification was achieved through biotin-streptavidin-mediated pull-down, followed by PCR amplification. The resulting Hi-C libraries were quantified and sequenced on the MGI-seq platform (BGI, China). Hi-C data analysis was conducted using the HiCPro pipeline. Reads were aligned to the UCSC mm10 reference genome using Bowtie2. Only reads with a MAPQ value greater than 10 were selected and assigned to MboI restriction fragments to construct interaction pairs. Invalid pairings were removed, and the remaining valid pairings were used to create the interaction matrix, which was normalized using the ICE method. Valid pairs were converted to the HiC format using Juicertools for loop analysis. Interaction matrices at various resolutions were utilized to identify topologically associating domains (TADs) or generate heatmaps.

TAD

TAD analysis was conducted utilizing HiCPro matrices and cworld insulation score analysis. Specifically, the 40kb resolution matrix generated by HiCPro was processed using a density extraction tool to obtain a sparse matrix. This sparse matrix was subsequently analyzed with cworld's matrix2insulation.pl script to calculate insulation scores. These scores were then used in conjunction with the insulation2tads tool to identify TAD boundaries. All parameters were set to

their default values throughout the process. To classify TADs from different samples, a custom Perl script was employed. The classification involved comparing TAD boundaries between treatment and control groups. Changes in TADs were assessed based on the interval from the start of the first matched boundary to the end of the first matched boundary. These changes were categorized into five distinct types: No Change, Separation, Fusion, Shift, and Cliques.

Loop

Loops were identified using Hiccups from Juicertools with resolution parameters set to 5,000, 10,000, and 25,000 base pairs and the normalization method set to KR, while all other parameters were kept at default values. The loops from all samples were merged into a comprehensive "all loop" set using Bedtools pairtopair. Loops larger than 1 megabase (Mb) were classified as long-range loops. Inter-chromatin interactions were specifically identified using the FitHiC algorithm, which detects contacts between two anchors located on different chromosomes. For the Dux overexpression (OE) sample, loop-associated CTCF reads were quantified using Bedtools multicov, and CTCF-enhanced loops were analyzed using DESeq2. Bedtools pairtobed was utilized to identify loops associated with super-enhancer regions. Genes directly contacted by loops were identified using Bedtools pairtobed and subsequently analyzed for gene expression levels. APA analysis was conducted using the R package GENOVA with parameters set to -k KR and -r 5000, generating heatmaps and calculating the ratio of observed-to-expected (Obs/Exp) values of central pixels relative to the average of the nine pixels in the lower-left corner, reflecting changes in loop interaction strength. Interaction frequencies of loops were extracted using StrawR, and a custom script was employed to calculate the Obs/Exp values for each loop, which were used to create boxplots and display loop heatmaps. DeepTools computeMatrix was used to calculate the enrichment of CTCF, P300, and Dux at loop anchors. Motif enrichment at loop anchors was assessed using HOMER. Hi-C data and other sequencing data were visualized using pyGenomeTracks.

References

CORCES, M. R., TREVINO, A. E., HAMILTON, E. G., GREENSIDE, P. G., SINNOTT-ARMSTRONG, N. A., VESUNA, S., SATPATHY, A. T., RUBIN, A. J., MONTINE, K. S., WU, B., KATHIRIA, A., CHO, S. W., MUMBACH, M. R., CARTER, A. C., KASOWSKI, M., ORLOFF, L. A., RISCA, V. I., KUNDAJE, A., KHAVARI, P. A., MONTINE, T. J., GREENLEAF, W. J. & CHANG, H. Y. 2017. An improved ATAC-seq protocol reduces background and enables interrogation of frozen tissues. *Nature Methods,* 14, 959-962.

HNISZ, D., ABRAHAM, B. J., LEE, T. I., LAU, A., SAINT-ANDRÉ, V., SIGOVA, A. A., HOKE, H. A. & YOUNG, R. A. 2013. Super-enhancers in the control of cell identity and disease. *Cell,* 155**,** 934-947.

LIU, X., JIANG, S., MA, L., QU, J., ZHAO, L., ZHU, X. & DING, J. 2021. Time-dependent effect of 1,6-hexanediol on biomolecular condensates and 3D chromatin organization. *Genome Biology,* 22**,** 230.

RAO, S. S. P., HUNTLEY, M. H., DURAND, N. C., STAMENOVA, E. K., BOCHKOV, I. D., ROBINSON, J. T., SANBORN, A. L., MACHOL, I., OMER, A. D., LANDER, E. S. & AIDEN, E. L. 2014. A 3D map of the human genome at kilobase resolution reveals principles of chromatin looping. *Cell,* 159**,** 1665-1680.

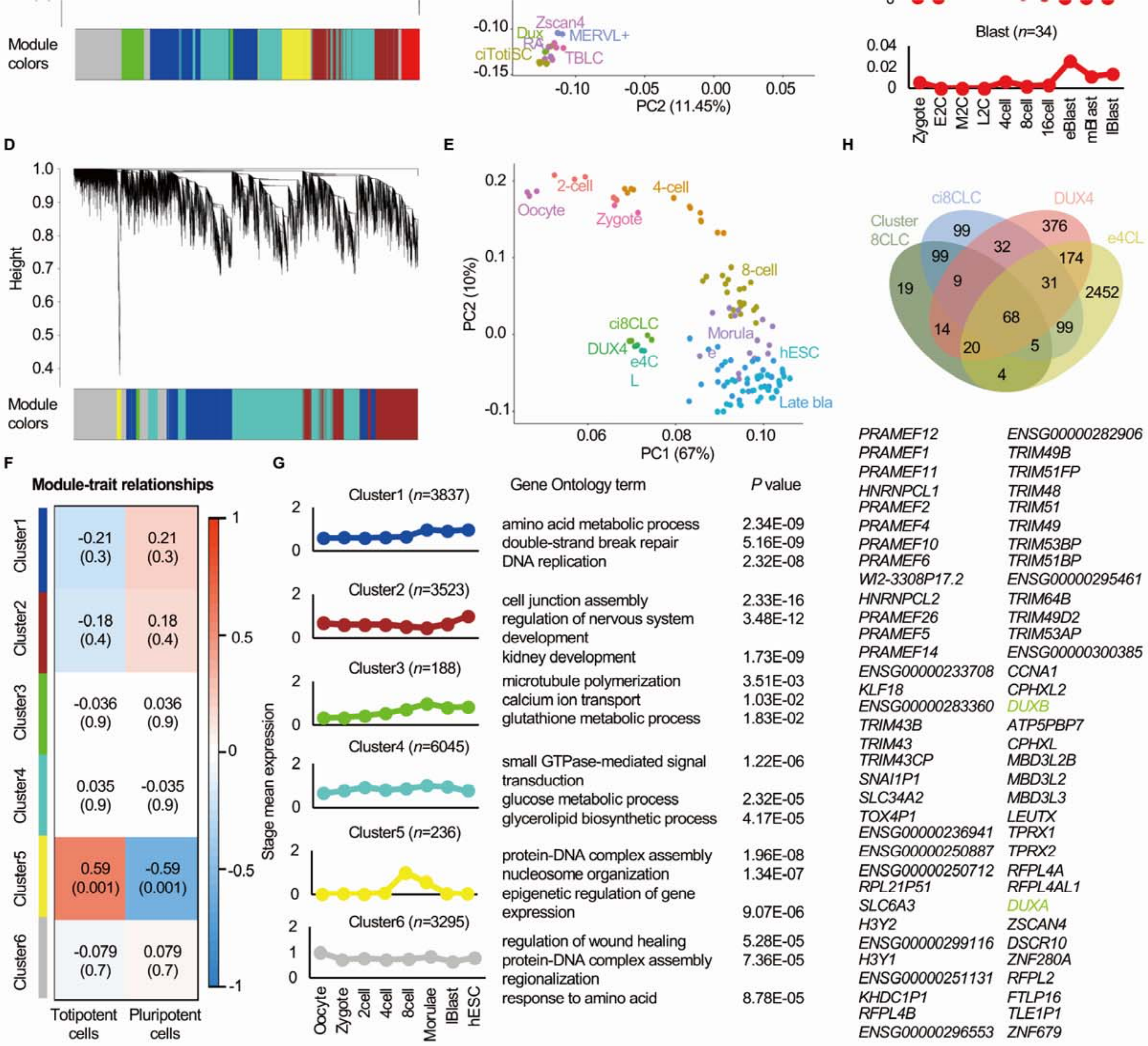
Module colors
Zscan4
Dux
MERVL+
RA
ciTotiSC
TBLC
-0.10
-0.15
-0.10 -0.05 0.00 0.05
PC2 (11.45%)
Blast (n=34)
0.04
0.02
0
Zygote E2C M2C L2C 4cell 8cell 16cell eBlast mBlast lBlast
D
1.0 0.9 0.8 0.7 0.6 0.5 0.4
Height
Module colors
E
0.2 0.1 0.0 -0.1
PC2 (10%)
0.06 0.08 0.10
PC1 (67%)
2-cell
Oocyte
Zygote
4-cell
8-cell
ci8CLC
DUX4
e4C
L
Morula
e
hESC
Late bla
H
ci8CLC
DUX4
Cluster 8CLC
e4CL
99 376 99 32 174 9 31 2452 19 68 99 14 20 5 4
F
Module-trait relationships
Cluster1 -0.21 (0.3) 0.21 (0.3)
Cluster2 -0.18 (0.4) 0.18 (0.4)
Cluster3 -0.036 (0.9) 0.036 (0.9)
Cluster4 0.035 (0.9) -0.035 (0.9)
Cluster5 0.59 (0.001) -0.59 (0.001)
Cluster6 -0.079 (0.7) 0.079 (0.7)
Totipotent cells
Pluripotent cells
1 0.5 0 -0.5 -1
G
Stage mean expression
Cluster1 (n=3837)
Cluster2 (n=3523)
Cluster3 (n=188)
Cluster4 (n=6045)
Cluster5 (n=236)
Cluster6 (n=3295)
Oocyte Zygote 2cell 4cell 8cell Morulae lBlast hESC
Gene Ontology term P value
amino acid metabolic process 2.34E-09
double-strand break repair 5.16E-09
DNA replication 2.32E-08
cell junction assembly 2.33E-16
regulation of nervous system development 3.48E-12
kidney development 1.73E-09
microtubule polymerization 3.51E-03
calcium ion transport 1.03E-02
glutathione metabolic process 1.83E-02
small GTPase-mediated signal transduction 1.22E-06
glucose metabolic process 2.32E-05
glycerolipid biosynthetic process 4.17E-05
protein-DNA complex assembly 1.96E-08
nucleosome organization 1.34E-07
epigenetic regulation of gene expression 9.07E-06
regulation of wound healing 5.28E-05
protein-DNA complex assembly 7.36E-05
regionalization
response to amino acid 8.78E-05
PRAMEF12
PRAMEF1
PRAMEF11
HNRNPCL1
PRAMEF2
PRAMEF4
PRAMEF10
PRAMEF6
WI2-3308P17.2
HNRNPCL2
PRAMEF26
PRAMEF5
PRAMEF14
ENSG00000233708
KLF18
ENSG00000283360
TRIM43B
TRIM43
TRIM43CP
SNAI1P1
SLC34A2
TOX4P1
ENSG00000236941
ENSG00000250887
ENSG00000250712
RPL21P51
SLC6A3
H3Y2
ENSG00000299116
H3Y1
ENSG00000251131
KHDC1P1
RFPL4B
ENSG00000296553
ENSG00000282906
TRIM49B
TRIM51FP
TRIM48
TRIM51
TRIM49
TRIM53BP
TRIM51BP
ENSG00000295461
TRIM64B
TRIM49D2
TRIM53AP
ENSG00000300385
CCNA1
CPHXL2
DUXB
ATP5PBP7
CPHXL
MBD3L2B
MBD3L2
MBD3L3
LEUTX
TPRX1
TPRX2
RFPL4A
RFPL4AL1
DUXA
ZSCAN4
DSCR10
ZNF280A
RFPL2
FTLP16
TLE1P1
ZNF679

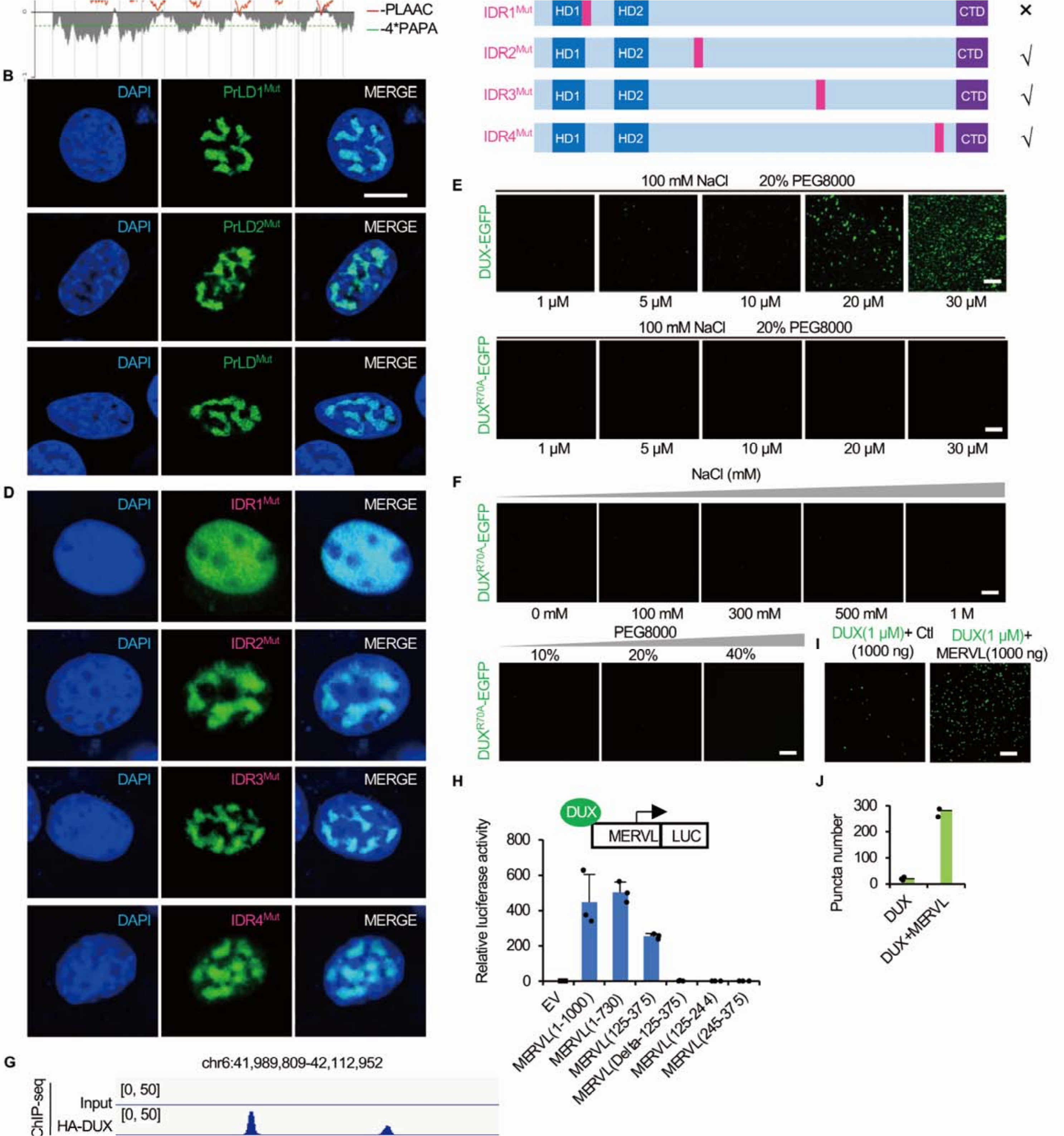
-PLAAC
-4*PAPA
B
DAPI
PrLD1Mut
MERGE
PrLD2Mut
PrLDMut
D
IDR1Mut
IDR2Mut
IDR3Mut
IDR4Mut
HD1
HD2
CTD
×
√
E
100 mM NaCl 20% PEG8000
DUX-EGFP
DUXR70A-EGFP
1 μM
5 μM
10 μM
20 μM
30 μM
F
NaCl (mM)
0 mM
100 mM
300 mM
500 mM
1 M
PEG8000
10%
20%
40%
I
DUX(1 μM)+ Ctl (1000 ng)
DUX(1 μM)+ MERVL(1000 ng)
H
DUX
MERVL
LUC
Relative luciferase activity
800
600
400
200
0
EV
MERVL(1-1000)
MERVL(1-730)
MERVL(125-375)
MERVL(Delta-125-375)
MERVL(125-244)
MERVL(245-375)
J
Puncta number
300
200
100
0
DUX
DUX+MERVL
G
chr6:41,989,809-42,112,952
ChIP-seq
Input
HA-DUX
[0, 50]

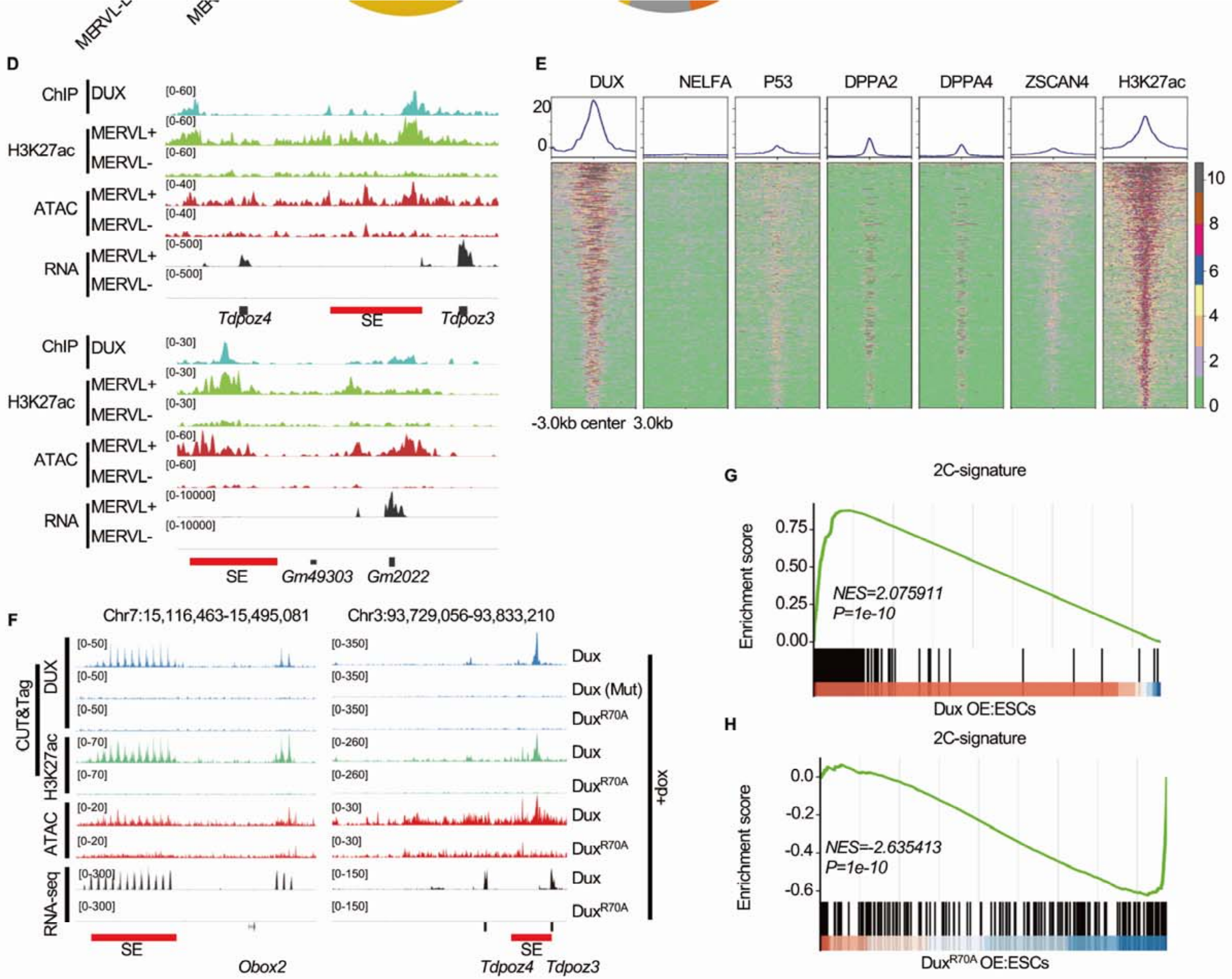

D
ChIP DUX
H3K27ac MERVL+ MERVL-
ATAC MERVL+ MERVL-
RNA MERVL+ MERVL-
Tdpoz4
SE
Tdpoz3
Gm49303
Gm2022
E
DUX
NELFA
P53
DPPA2
DPPA4
ZSCAN4
H3K27ac
-3.0kb center 3.0kb
F
Chr7:15,116,463-15,495,081
Chr3:93,729,056-93,833,210
CUT&Tag
DUX
H3K27ac
ATAC
RNA-seq
Dux
Dux (Mut)
Dux^R70A
+dox
Obox2
G
2C-signature
Enrichment score
NES=2.075911
P=1e-10
Dux OE:ESCs
H
2C-signature
Enrichment score
NES=-2.635413
P=1e-10
Dux^R70A OE:ESCs

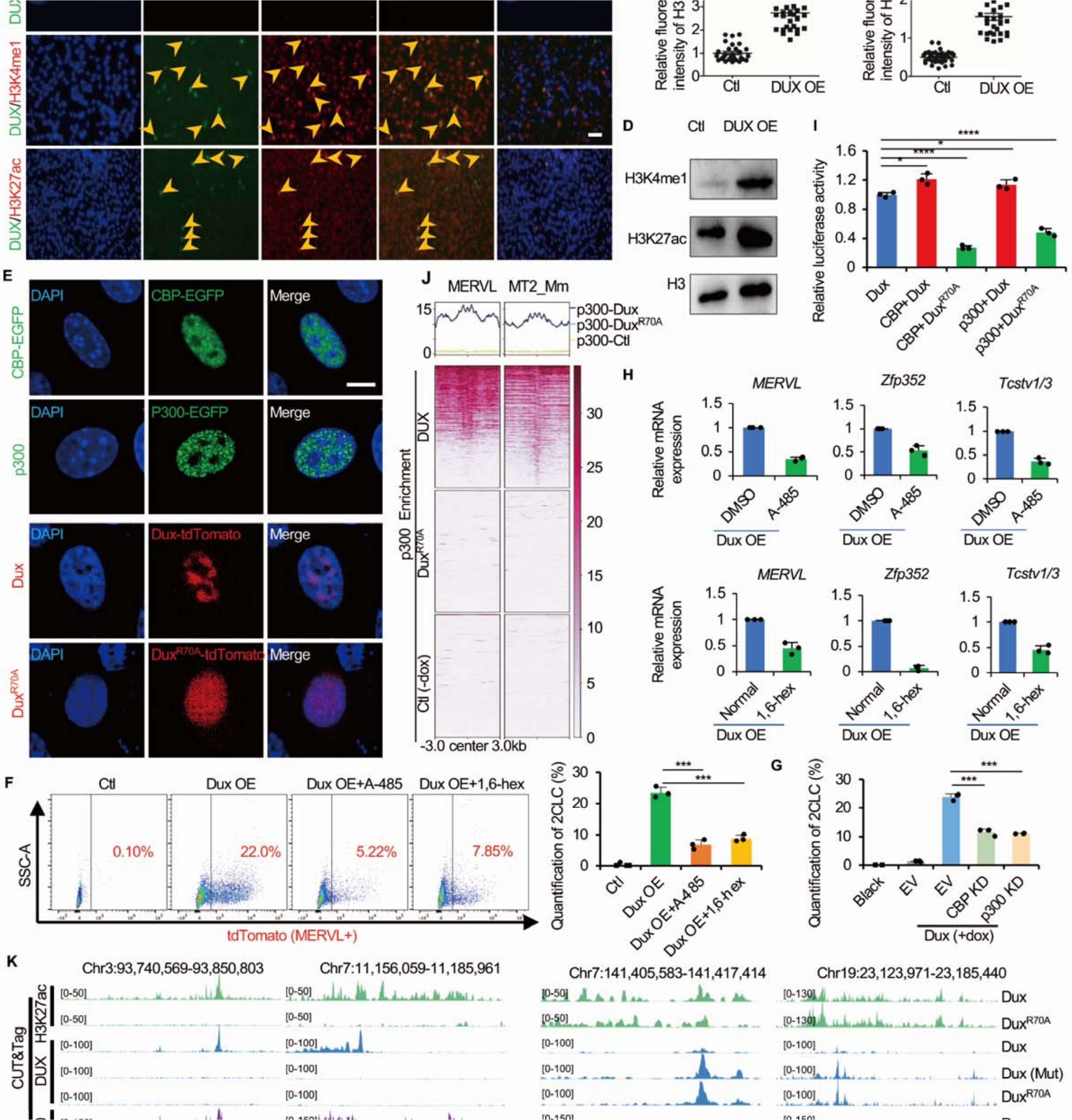

DUX/H3K4me1
DUX/H3K27ac
Relative fluore intensity of H3
Relative fluor intensity of H
Ctl
DUX OE
D
Ctl DUX OE
H3K4me1
H3K27ac
H3
I
Relative luciferase activity
Dux
CBP+ Dux
CBP+ Dux^{R70A}
p300+ Dux
p300+Dux^{R70A}
E
DAPI
CBP-EGFP
Merge
P300-EGFP
p300
Dux-tdTomato
Dux
Dux^{R70A}-tdTomato
Dux^{R70A}
J
MERVL
MT2_Mm
p300-Dux
p300-Dux^{R70A}
p300-Ctl
p300 Enrichment
DUX
Dux^{R70A}
Ctl (-dox)
-3.0 center 3.0kb
H
MERVL
Zfp352
Tcstv1/3
Relative mRNA expression
DMSO
A-485
Dux OE
Normal
1,6-hex
F
Ctl
Dux OE
Dux OE+A-485
Dux OE+1,6-hex
0.10%
22.0%
5.22%
7.85%
SSC-A
tdTomato (MERVL+)
Quantification of 2CLC (%)
Dux OE+A-485
Dux OE+1,6-hex
G
Black
EV
CBP KD
p300 KD
Dux (+dox)
K
Chr3:93,740,569-93,850,803
Chr7:11,156,059-11,185,961
Chr7:141,405,583-141,417,414
Chr19:23,123,971-23,185,440
CUT&Tag
H3K27ac
DUX
Dux
Dux^{R70A}
Dux (Mut)

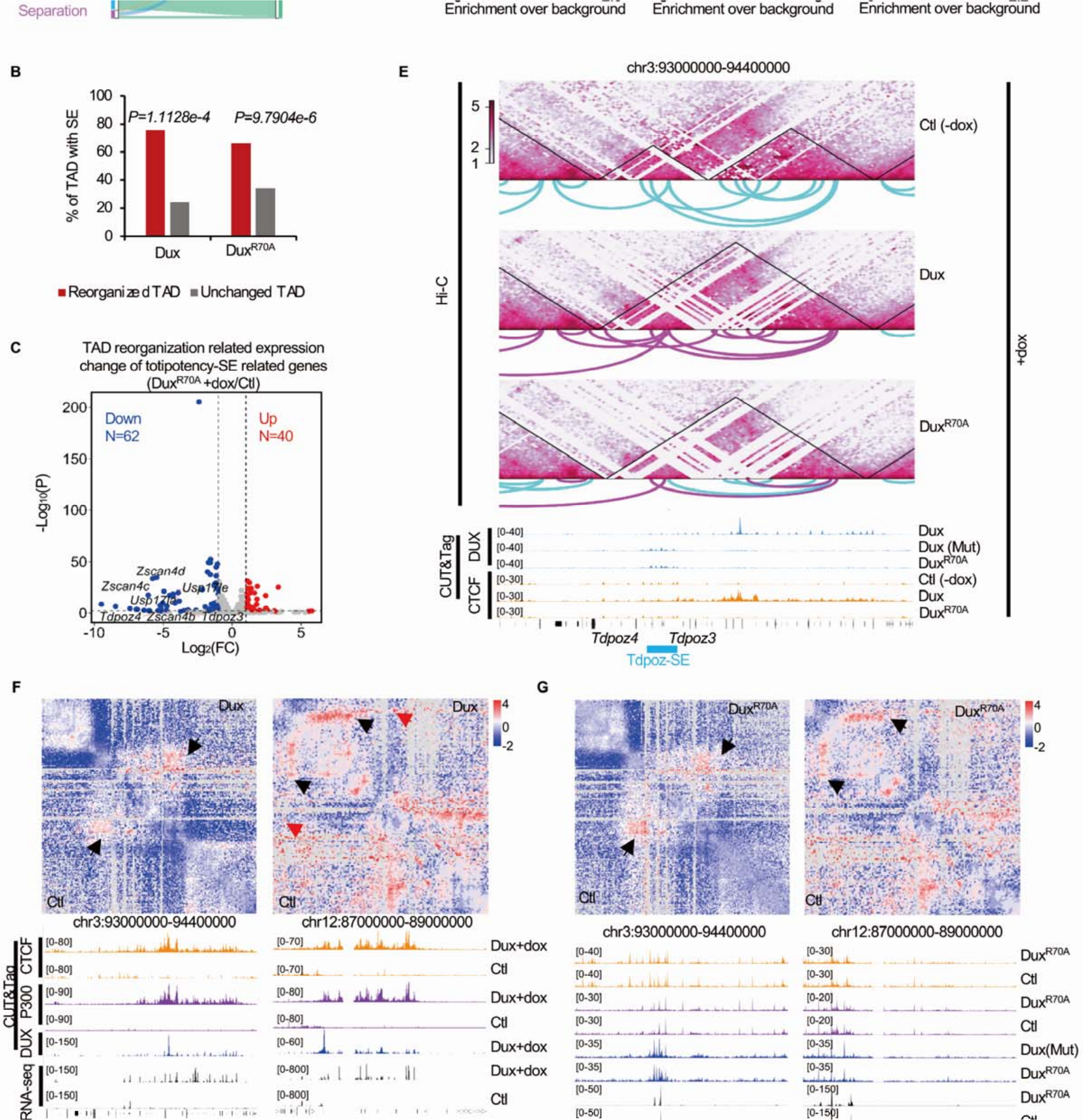
Separation
Enrichment over background
Enrichment over background
Enrichment over background
B
P=1.1128e-4
P=9.7904e-6
% of TAD with SE
Dux
DuxR70A
Reorganized TAD
Unchanged TAD
C
TAD reorganization related expression change of totipotency-SE related genes (DuxR70A +dox/Ctl)
Down N=62
Up N=40
-Log10(P)
Log2(FC)
Zscan4d
Zscan4c
Usp17la
Usp17le
Tdpoz4
Zscan4b
Tdpoz3
E
chr3:93000000-94400000
Hi-C
Ctl (-dox)
Dux
DuxR70A
+dox
CUT&Tag
DUX
CTCF
Dux (Mut)
Tdpoz4
Tdpoz3
Tdpoz-SE
F
chr3:93000000-94400000
chr12:87000000-89000000
Dux+dox
Ctl
P300
RNA-seq
G
DuxR70A
Dux(Mut)

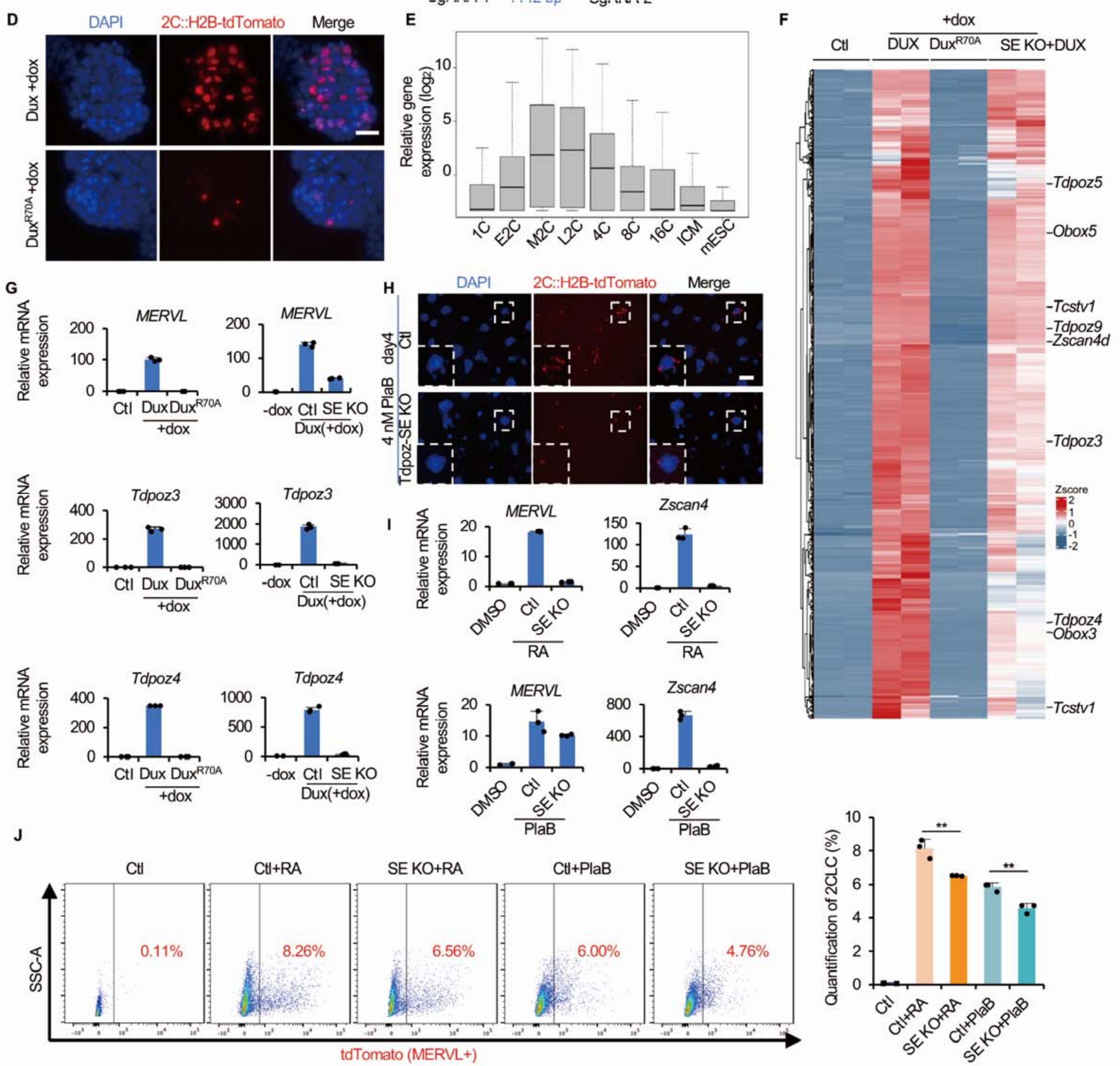
D
DAPI
2C::H2B-tdTomato
Merge
Dux +dox
Dux^R70A +dox
E
Relative gene expression (log2)
10
5
0
1C
E2C
M2C
L2C
4C
8C
16C
ICM
mESC
F
+dox
Ctl
DUX
Dux^R70A
SE KO+DUX
Tdpoz5
Obox5
Tcstv1
Tdpoz9
Zscan4d
Tdpoz3
Zscore
2
1
0
-1
-2
Tdpoz4
Obox3
Tcstv1
G
Relative mRNA expression
MERVL
200
100
0
Ctl
Dux
Dux^R70A
+dox
MERVL
200
100
0
-dox
Ctl
SE KO
Dux(+dox)
Tdpoz3
400
200
0
Ctl
Dux
Dux^R70A
+dox
Tdpoz3
3000
2000
1000
0
-dox
Ctl
SE KO
Dux(+dox)
Tdpoz4
400
200
0
Ctl
Dux
Dux^R70A
+dox
Tdpoz4
1000
500
0
-dox
Ctl
SE KO
Dux(+dox)
H
DAPI
2C::H2B-tdTomato
Merge
day4
Ctl
4 nM PlaB
Tdpoz-SE KO
I
Relative mRNA expression
MERVL
20
10
0
DMSO
Ctl
SE KO
RA
Zscan4
150
100
50
0
DMSO
Ctl
SE KO
RA
MERVL
20
10
0
DMSO
Ctl
SE KO
PlaB
Zscan4
800
400
0
DMSO
Ctl
SE KO
PlaB
J
Ctl
Ctl+RA
SE KO+RA
Ctl+PlaB
SE KO+PlaB
SSC-A
0.11%
8.26%
6.56%
6.00%
4.76%
tdTomato (MERVL+)
Quantification of 2CLC (%)
10
8
6
4
2
0
**
**
Ctl
Ctl+RA
SE KO+RA
Ctl+PlaB
SE KO+PlaB

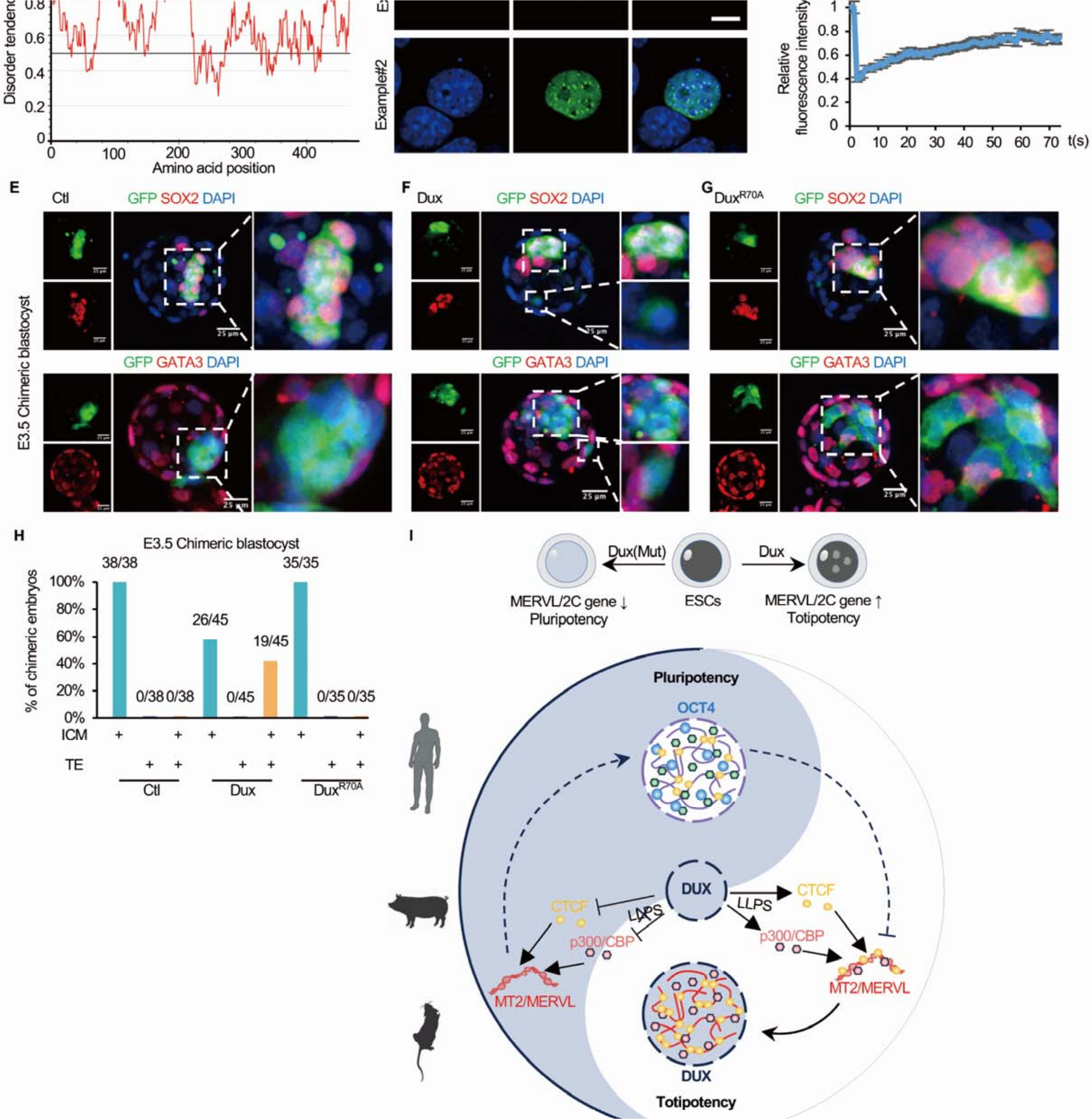
Disorder tendency
0.8
0.6
0.4
0.2
0
0
100
200
300
400
Amino acid position
Example#2
Relative fluorescence intensity
1
0.8
0.6
0.4
0.2
0
0
10
20
30
40
50
60
70
t(s)
E
Ctl
GFP SOX2 DAPI
F
Dux
GFP SOX2 DAPI
G
DuxR70A
GFP SOX2 DAPI
E3.5 Chimeric blastocyst
25 μm
GFP GATA3 DAPI
GFP GATA3 DAPI
GFP GATA3 DAPI
H
E3.5 Chimeric blastocyst
% of chimeric embryos
100%
80%
60%
40%
20%
0%
38/38
0/38
0/38
26/45
0/45
19/45
35/35
0/35
0/35
ICM
TE
Ctl
Dux
DuxR70A
I
Dux(Mut)
Dux
MERVL/2C gene ↓
Pluripotency
ESCs
MERVL/2C gene ↑
Totipotency
Pluripotency
OCT4
DUX
CTCF
LLPS
p300/CBP
MT2/MERVL
CTCF
LLPS
p300/CBP
MT2/MERVL
DUX
Totipotency

Supplementary data

Phase Separation of DUX Family Proteins Drives Totipotency-like State via 3D Genome Reorganization and Retrotransposon Activation

Leilei Gao[1,3,4,10], Qifeng Gao[1,3,4,10], Na Hai[7,11], Ziqiang Wu[5,11], Penghui Li[5,11], Han Kang[1,3,4], Xiaohui Song[1,3,4], Jinlian Hua[1,3,4], Shiqiang Zhang[1,3,4], Gang Ren[4,8], Jihong Yang[9], Leqian Yu[6,*], Yulei Wei[7,*], Junjun Ding[5,*], Fan Yang[1,2,3,4,12,*]

Supplemental figure legends

Fig. S1. Analysis totipotent related hub genes.

(A) Hierarchical clustering tree of pluripotent cells and totipotent cells (MERVL+, Dux OE, Zscan4c OE, TBLC, ciTotiSC).

(B) Principal Component Analysis (PCA) of totipotent cells and mouse preimplantation embryos utilizing transcriptomic datasets.

(C) Hub genes associated with totipotency, as depicted in Figure 1C, exhibit distinct stage-specific expression patterns during mouse pre-implantation development.

(D) Hierarchical clustering dendrogram of pluripotent and totipotent cells, including ci8CLC, LEUTX+, DUX4-overexpressing cells, and e4CL.

(E) PCA of totipotent cells and human preimplantation embryos based on transcriptomic profiling.

(F) Module-trait relationship analysis. The values in each cell represent the correlation coefficients between module eigengenes and sample traits. Corresponding p-values for each module are provided in parentheses.

(G) Six clusters of genes exhibit distinct stage-specific expression patterns during human pre-implantation development. The right panel displays representative enriched Gene Ontology terms along with their corresponding p-values.

(H) Venn diagram illustrating those 68 genes from cluster 5, as shown in Figure S1F, were concurrently upregulated across multiple models of totipotent cells, including ci8CLC, DUX4 overexpression and e4CL.

Fig. S2. Identification of the region in IDR required for DUX phase separation.

(A) The analysis of prion-like domain (PrLD) distribution within DUX using PLAAC identified two PrLDs, which are indicated by green arrows. "Background" refers to a region within the DUX protein that was predicted to have low prion-like propensity using the PLAAC algorithm (http://plaac.wi.mit.edu/), which compares amino acid composition against a proteome-derived background model.

(B) Live-cell images illustrating DUX in the absence of a PrLD. Scale bars: 5 μm.

(C) An overview of the DUX mutants utilized to identify the IDRs and PrLDs responsible for phase separation.

(D) Live-cell images illustrating DUX in the absence of an IDR. Scale bars: 5 μm.

(E) LLPS of purified recombinant EGFP-DUX with IDR^{Wt} (top) and IDR^{Mut} (bottom) at increasing concentrations under 20% PEG8000 and 100 mM NaCl condition.

(F) Condensates of purified recombinant $EGFP\text{-}DUX^{R70A}$ under various concentrations NaCl (top) or PEG8000 (bottom).

(G) Schematic representation of the full-length or truncated MERVL LTR fragments in the luciferase reporter vector.

(H) Dual luciferase assay for detecting MERVL truncations activated by DUX.

(I) Representative fluorescence microscopy images depicting the aggregation of EGFP-DUX following the addition of MERVL short sequence. The concentration of EGFP-DUX was maintained at 1 μM. The mass of MERVL was 1 μg.

(J) Quantification of the puncta number of EGFP-DUX formed with MERVL DNA. Data are presented as the mean ± SEM. ****$P < 0.0001$ (t-test).

Fig. S3. DUX expression is essential for the SEs establishment in the totipotent cells.

(A) Distributions of the totipotency-related TEs and SEs on MERVL/LTR.

(B) Genomic distributions of totipotency-associated TEs and SEs.

(C) Comparison of SEs and TEs in terms of number, length, and enriched H3K27ac reads.

(D) Integrative Genomics Viewer (IGV) displaying DUX ChIP-seq, H3K27ac ChIP-seq, ATAC-seq, and RNA-seq data for MERVL-positive and MERVL-negative samples at two representative top-ranked super-enhancer loci. The positions of the super-enhancers are highlighted by bold red lines.

(E) Heatmaps depicting the ChIP-seq signal intensity of DUX, NELFA, p53, DPPA2/4, ZSCAN4, and the histone modification H3K27ac in the genomic regions surrounding totipotency-associated super-enhancers.

(F) Representative tracks for the key 2C genes activated by the LLPS of DUX.

(G-H) Gene set enrichment analysis (GSEA) of specific genes expressed in 2-cell in Dux overexpressing (G) and Dux^{R70A} overexpressing ESCs (H).

Fig. S4. Dux LLPS deficiency fails to activate H3K27 acetylation as well as the genes associated with super-enhancers.

(A) Immunostaining analysis of Dux-induced cells revealed an increase in H3K27ac and H3K4me1 histone modifications. Arrows indicate the enrichment of histone modifications in cells overexpressing Dux (n = 3 biological replicates). Scale bars: top, 5 µm; bottom, 50 µm.

(B) Quantification of the fluorescence intensity along the line embedded within the puncta, as indicated by the arrow in Figure S4A.

(C) Statistical analysis of the relative fluorescent intensity of H3K27ac and H3K4me1 following Dux overexpression. Data are represented as the mean ± SEM. Statistical significance was determined using a two-tailed unpaired t-test, with **$P < 0.01$**.

(D) Western blotting analysis was conducted to examine the levels of H3K27ac, H3K4me1, and H3K4me3 in both control (Ctl) and DUX overexpression cells.

(E) Fluorescence microscopy images of tdTomato-p300, EGFP-CBP, tdTomato tagged-DUX with either IDR^{Wt} or IDR^{Mut} expressed individually in the cells ($n = 3$ biological replicates). Scale bars: 5 µm.

(F) Flow cytometry profiles of MERVL-positive cells following treatment with A-485 or 1.6-Hex, as shown in Figure 4D.

(G) Flow cytometry analysis of the percentage of MERVL-positive cells induced by Dux upon CBP/p300 knockdown.

(H) RT-qPCR analysis of *MERVL*, *Zfp352*, *Tcstv1/3* expression following Dux overexpression in the presence of A-485 or 1,6-Hexanediol, respectively. Data are represented as mean ± SD.

(I) Dual luciferase assay analysis was conducted to evaluate MERVL expression activated by Dux or Dux^{R70A} in the presence of co-transfection with p300/CBP, respectively (n = 3 biological replicates). Data are presented as the mean ± SEM. *$P < 0.05$; ****$P < 0.0001$ (t-test).

(J) Heatmap showing enrichment of p300 on MERVL/MT2_Mm in Dux-expressing and Dux^{R70A}-expressing cells.

(K) Representative tracks for the H3K27ac modification and RNA expression change of 2C gene indued by p300 in a Dux-dependent manner (left). Representative tracks illustrating gene loci bound by Dux^{R70A} but not activated following Dux^{R70A} expression (right).

Fig. S5. IDRs of DUX induce totipotency related TAD-reorganization.

(A) Sankey diagrams illustrating the reorganization of TADs in Dux-expressing cells.

(B) The distribution percentage of the TADs with SEs.

(C) Volcano plot illustrating the expression changes of totipotency-SE associated genes related to TAD reorganization in cells overexpressing Dux^{R70A}. Gray dots represent genes with no significant change in expression; red dots indicate upregulated genes; blue dots signify downregulated genes. The majority of SE-associated genes exhibited upregulation following TAD reorganization in Dux-overexpressing cells (right panel), as opposed to those overexpressing DUX^{R70A}.

(D) Homer motif enrichment analysis of chromatin loops in control (Ctl) ESCs (left), DUX-overexpressing ESCs (middle), and Dux^{R70A}-overexpressing ESCs (right).

(E) Representative TADs and chromatin loops were visualized in control (Ctl), Dux-overexpressing, and Dux^{R70A}-overexpressing ESCs. Blue arcs denote the original loops present in the control group, while pink arcs highlight the newly formed loops following Dux or Dux^{R70A} overexpression.

(F-G) In Dux-expressing cells (above diagonal) and control cells (below diagonal), Hi-C detected enhanced loops (left, indicated by black arrow) and newly generated Dux-associated loops (right, indicated by red arrow). The bottom panels illustrate CTCF (orange), p300 (purple), and DUX (blue) CUT&Tag signals, as well as RNA-seq data in both control (Ctl) and Dux-overexpressing ESCs (F). The loci depicted in Figure S5G were detected by Hi-C in ESCs with Dux^{R70A} expressing (above the diagonal) and the control (below the diagonal) (G).

Fig. S6. Tdpoz-SE knockout restricts the conversion of 2CLCs.

(A) Venn diagram illustrating the intersection of totipotency-related SEs activated by Dux but not Dux^{R70A}.

(B) The strategy to knock out Tdpoz-SEs in ESCs by CRISPR-Cas9.

(C) Fluorescence microscope images depict MERVL-positive cells following Dux overexpression in Tdpoz-SE knockout cells (*n*=3 biological replicates). Scale bars: 50 µm.

(D) Fluorescence microscope images showing the MERVL positive cells after overexpressing Dux and LLPS deficiency DUX^{R70A} (*n* = 3 biological replicates). Scale bars: 50 µm.

(E) Box-and-whisker plot illustrating the normalized fold change (FC) expression levels of genes downregulated in Tdpoz-SE knockout cells compared to Dux overexpressing ESCs (Figure 6F). The center line represents the median; the box limits indicate the upper and lower quartiles; the whiskers extend to 1.5 times the interquartile range. ICM denotes the inner cell mass.

(F) Heatmap showing genes downregulated in Tdpoz-SE knockout cells compared to Dux overexpressing ESCs (n = 2 biological replicates).

(G) Relative expression levels of *MERVL*, *Tdpoz3*, *Tdpoz4* upon overexpression of Dux in the Tdpoz-SE knockout cells. RT-qPCR data are presented as average±SD.

(H) Fluorescence microscopy images showing MERVL-positive cells in Tdpoz-SE knockout cells after treatment with 4 nM PlaB for 4 days (*n* = 3 biological replicates). Scale bars: 50 µm.

(I) Relative expression levels of *MERVL*, *Tdpoz3*, *Tdpoz4* in the Tdpoz-SE knockout cells after treatment with 0.53 µM RA for 48 h or 4 nM PlaB for 4 days. RT-qPCR data are presented as average±SD.

(J) Flow cytometry profiles of MERVL-positive cells following treatment with RA or PlaB, as shown in Figure 6G and Figure S6H.

Fig. S7. The expanded developmental potency of Dux-activated cells is LLPS-dependent.

(A) Alignment of the DUX protein homeodomain across murine, rat, human and porcine species.

(B) Protein sequence analysis and disorder prediction (using IUPred) for the DUXC. The numbers above the sequence represent the positions of the amino acids (aa). A score exceeding 0.5 indicates regions predicted to be disordered.

(C) Live-cell imaging of cells expressing EGFP-DUXC. Scale bar: 5 µm.

(D) FRAP analysis of EGFP-DUXC droplets in the cells was conducted by bleaching at positions indicated by white arrows. Quantification of fluorescence intensity during the FRAP assay is shown below ($n = 7$ cells). Scale bars: 5 µm.

(E-G) Immunofluorescence staining was performed on E3.5 blastocysts. SOX2 and GATA3 served as markers for the inner cell mass (ICM) and trophectoderm (TE), respectively. Nuclei were counterstained with DAPI (blue). Scale bars: 25 µm.

(H) The proportion of embryos and the number of chimeric embryos exhibiting contributions to the trophectoderm (TE), inner cell mass (ICM), and both TE and ICM at the E3.5 stage.

(I) The mechanism pattern diagram of DUX LLPS regulating the transition from pluripotency to totipotency is different from the way OCT4 is regulated.

Video S1-3. Live-cell imaging showing the sequential formation of DUX condensates followed by MERVL activation, related to Figure 2.

Table S1A. List of samples used for WGCNA analysis of mouse totipotent states

GSE accession	GSM accession	original sample annotation	state
GSE185005	GSM5603033	ciTotiSC-P1	Totipotent
GSE185005	GSM5603034	ciTotiSC-P1	Totipotent
GSE185005	GSM5603035	ciTotiSC-P2	Totipotent
GSE185005	GSM5603036	ciTotiSC-P2	Totipotent
GSE185005	GSM5603037	ciTotiSC-P3	Totipotent
GSE185005	GSM5603038	ciTotiSC-P3	Totipotent
GSE185005	GSM5603039	ciTotiSC-P4	Totipotent
GSE185005	GSM5603040	ciTotiSC-P4	Totipotent
GSE185005	GSM5603041	OG	Pluripotent
GSE185005	GSM5603042	OG	Pluripotent
GSE168728	GSM5160094	TC1-EGFP-P10	Totipotent
GSE168728	GSM5160095	TC1-EGFP-PP15	Totipotent
GSE168728	GSM5160099	TC1-EGFP-PP5	Totipotent
GSE168728	GSM5160100	TC1-EGFP-PP6	Totipotent
GSE168728	GSM5160103	TC1-EGFP-PP7	Totipotent
GSE168728	GSM5160104	TBLC-P6	Totipotent
GSE168728	GSM5160101	TC1-EGFP-r1	Pluripotent
GSE168728	GSM5160102	TC1-EGFP-r2	Pluripotent
GSE222636	GSM6102043	Dux-dox24h	Totipotent
GSE222636	GSM6102040	Dux-dox24h	Totipotent
GSE222636	GSM6102031	Dux-dox0h	Pluripotent
GSE222636	GSM6102027	Dux-dox0h	Pluripotent
GSE114952	GSM3160746	E3.5-Zscan4oe	Totipotent
GSE114952	GSM3160754	E3.5	Pluripotent

Table S1B. List of samples used for WGCNA analysis of human totipotent states

Data source	accession Number	original sample annotation	state
GSE197641	SRR18181015	ci8CLC-r1	Totipotent
GSE197641	SRR18181016	ci8CLC-r2	Totipotent
GSE197641	SRR18181017	ci8CLC-r3	Totipotent
GSE197641	SRR18181018	ci8CLC-r4	Totipotent
GSE197641	SRR18181019	prEpiSC-r1	Pluripotent
GSE197641	SRR18181020	prEpiSC-r2	Pluripotent
GSE197641	SRR18181021	LEUTX-mCherry+	Totipotent
GSE197641	SRR18181022	LEUTX-mCherry-	Pluripotent
https://ftp.cngb.org/pub/CNSA/data1/CNP0001454/	CNS0305434	H9_e4CL_D5	Totipotent
https://ftp.cngb.org/pub/CNSA/data1/CNP0001454/	CNS0305435	H9_e4CL_D5	Totipotent
https://ftp.cngb.org/pub/CNSA/data1/CNP0001454/	CNS0305430	H9_4CL_P5	Pluripotent
https://ftp.cngb.org/pub/CNSA/data1/CNP0001454/	CNS0305431	H9_4CL_P5	Pluripotent
https://ftp.cngb.org/pub/CNSA/data1/CNP0001454/	CNS0305432	H9_Primed	Pluripotent
https://ftp.cngb.org/pub/CNSA/data1/CNP0001454/	CNS0305433	H9_Primed	Pluripotent
GSE85632	GSM2515763	Luciferase_14hrs_rep1	Pluripotent
GSE85632	GSM2515764	Luciferase_14hrs_rep2	Pluripotent
GSE85632	GSM2515765	Luciferase_24hrs_rep1	Pluripotent
GSE85632	GSM2515766	Luciferase_24hrs_rep2	Pluripotent
GSE85632	GSM2515767	DUX4CA_14hrs_rep1	Totipotent
GSE85632	GSM2515768	DUX4CA_14hrs_rep2	Totipotent
GSE85632	GSM2515769	DUX4CA_24hrs_rep1	Totipotent
GSE85632	GSM2515770	DUX4CA_24hrs_rep2	Totipotent

Table S2. Primers used in this paper

Name	Orientation	Sequence	Use for
MERVL probe	F	CAGATTGAATCTACTAGATTGAATCTACTAGATTGAATCT	EMSA
	R	AGATTCAATCTAGTAGATTCAATCTAGTAGATTCAATCT	
Tdpoz-SE left	F	CACCgCTACTCTTGTGGATCTTTCT	Tdpoz-SE knock out
	R	AAACAGAAAGATCCACAAGAGTAGC	
Tdpoz-SE right	F	CACCgAATGTGGCAGAGCTCCTATA	
	R	AAACTATAGGAGCTCTGCCACATTC	
CBP	shRNA1	TAACTCTGGCCATAGCTTAAT	CBP/p300 knockdown
	shRNA2	GCTATCAGAATAGGTATCATT	
P300	shRNA1	CCCTGGATTAAGTTTGATAAA	
	shRNA2	TACACTAGAGACACCTTGTAT	
Actin	F	TAGGCACCAGGGTGTGATGG	RT-qPCR
	R	CATGGCTGGGGTGTTGAAGG	
GAPDH	F	TGCACCACCAACTGCTTAGC	
	R	GGCATGGACTGTGGTCATGAG	
Hprt1	F	CTGGTGAAAAGGACCTCTCGAAG	
	R	CCAGTTTCACTAATGACACAAACG	
Rpl13a	F	CTGCTCTCAAGGTTGTTCGGCT	
	R	CCTTCCGTTTCTCCTCCAGAGT	
Tbp	F	CTACCGTGAATCTTGGCTGTAAAC	
	R	AATCAACGCAGTTGTCCGTGGC	
Ywhaz	F	CAGAAGACGGAAGGTGCTGAGA	
	R	CTTTCTGGTTGCGAAGCATTGGG	
Tdpoz1	F	CCTAGTTTTGCTCAGCCATCTG	
	R	ATAGTAGGCAATGAAATCAAGG	
CBP	F	TGCCAAGTTGCCCATTGTG	
	R	TTGTTGGTTTCGCTTGTCACT	
P300	F	TTCAAACGCCGAGTCTTCTT	
	R	GTTGAGCTGCTGTTGGCATA	
Zscan4	F	AAATGCCTTATGTCTGTTCCCTATG	
	R	TGTGGTAATTCCTCAGGTGACGAT	
Zfp352	F	TACGACGACCTGTCTCGG	
	R	TTCCTGGGTATGCTGTTG	
Tcstv1/3	F	GGATCCCTGAAGGTAAATCCTC	
	R	AACCATCCATCCTCAGGAAC	
Tdpoz3	F	GGGCACATCAGAAAAGTATTACC	
	R	GGCATGTCAAATACGGTTCCA	
Tdpoz4	F	TTGTTGAGGAAACGGAGGAAT	
	R	AATGCTGAAGGAGTCTTGG	
MERVL	F	AGGCTGCTCTACCACTTGGA	
	R	CAGGCCAAGAGCTGTTTTTC	
hActin	F	CATGTACGTTGCTATCCAGGC	
	R	CTCCTTAATGTCACGCACGAT	
hZscan4	F	TTTCAGTGTGAACCATCCGAG	
	R	AGCACCATTCTTGAGAACTCAG	
hLEUTX	F	AAGGAGGAGACTCCCTCAGC	
	R	AAAGAGAGTGGAGGCCCAAG	
hPRAMEF12	F	TCACCTCTCAGTTCCTCAAGC	
	R	CAGGCATTCGGTCATTACG	
hTRIM43	F	ACCCATCACTGGACTGGTGT	
	R	CACATCCTCAAAGAGCCTGA	

DUX4-CA	F	TTCGAGCGCAATCCCTATCC	
	R	CCCAGTTTCTCTGGCGAGTT	
